# Yes-associated protein contributes to magnesium alloy-derivedinflammation in endothelial cells

**DOI:** 10.1093/rb/rbac002

**Published:** 2022-01-20

**Authors:** Hongchi Yu, Zhe Hou, Nuoya Chen, Rifang Luo, Li Yang, Michael Miao, Xiaoyi Ma, Lifeng Zhou, Fugui He, Yang Shen, Xiaoheng Liu, Yunbing Wang

**Affiliations:** 1 National Engineering Research Center for Biomaterials, Sichuan University, Chengdu 610065, China; 2 Institute of Biomedical Engineering, West China School of Basic Medical Sciences & Forensic Medicine, Sichuan University, Chengdu 610041, China; 3 Division of Oral & Craniofacial Health Sciences, University of North Carolina Adams School of Dentistry, Chapel Hill, NC 27599, USA; 4 Beijing Key Laboratory of Cardiac Drug Device Technology and Evidence Based Medicine, Beijing 100021, China

**Keywords:** magnesium alloy, Yes-associated protein, inflammation

## Abstract

Magnesium alloy (Mg alloy) has attracted massive attention in the potential applications of cardiovascular stents because of its good biocompatibility and degradability. However, whether and how the Mg alloy induces inflammation in endothelial cells remains unclear. In the present work, we investigated the activation of Yes-associated protein (YAP) upon Mg alloy stimuli and unveiled the transcriptional function in Mg alloy-induced inflammation. Quantitative RT–PCR, western blotting and immunofluorescence staining showed that Mg alloy inhibited the Hippo pathway to facilitate nuclear shuttling and activation of YAP in human coronary artery endothelial cells (HCAECs). Chromatin immunoprecipitation followed sequencing was carried out to explore the transcriptional function of YAP in Mg alloy-derived inflammation. This led to the observation that nuclear YAP further bonded to the promoter region of inflammation transcription factors and co-transcription factors. This binding event activated their transcription and modified mRNA methylation of inflammation-related genes through regulating the expression of N6-methyladenosine modulators (*METTL3, METTL14, FTO* and* WTAP*). This then promoted inflammation-related gene expression and aggravated inflammation in HCAECs. In YAP deficiency cells, Mg alloy-induced inflammation was reduced. Collectively, our data suggest that YAP contributes to the Mg alloy-derived inflammation in HCAECs and may provide a potential therapeutic target that alleviates inflammation after Mg alloy stent implantation.

## Introduction

Coronary heart disease (CHD) counts for 43.2% of deaths attributable to cardiovascular disease, which is the most prevalent disease in the world [1]. Coronary stenting has become the most common catheter-based treatment for CHD due to improved clinical outcomes over percutaneous transluminal coronary angioplasty. Compared with permanent implantation materials, the biodegradable materials disappear entirely, thus avoiding some lifelong problems caused by permanent implants, including permanent physical irritation and local chronic inflammatory reactions [2]. Biodegradable metallic materials, including magnesium (Mg)-, iron (Fe)- and zinc (Zn)-based alloys, have an excellent combination of strength and ductility. Therefore, they are proposed for coronary stent implantation [3]. For the more daily intake dosage and similar biomechanical characters to natural tissues [4], Mg-based alloys have been successfully used in several clinical trials [5, 6].

After a stent implant, foreign body reaction drives activated platelets, monocytes, neutrophils and leukocytes to adhere to the injured endothelium, followed by macrophage accumulation [7]. Subsequently, smooth muscle cells and lymphocytes cover the stent struts to comprise a neointima [8]. Extensive studies from a materials perspective have focused on corrosion inhibition and improvement of mechanical properties of magnesium alloy (Mg alloy) and greatly advanced the clinical application of Mg alloy [9, 10]. Although some new bulk Mg alloy and surface coatings have a friendly role after implantation [11–13], the inflammatory response was examined by the phenotypic switching of macrophages in those studies. However, it is still far from clear whether and how the Mg alloy induces inflammation in endothelial cells. Endothelial cells are in direct contact with Mg alloy stent after implantation and are essential for maintaining the integrity of blood vessels by avoiding thrombosis and hyperplasia [14]. Therefore, exploring the role and the underlying mechanism by which Mg alloy induces inflammation in endothelial cells may show new light into the improvement of Mg alloys modification from the biological perspective.

Yes-associated protein (YAP) 1 and its paralog, TAZ, bind directly to the promoter region of target genes with transcriptional enhanced associate domain （TEAD） factors in the chromatin loop [15]. It can be activated by mechanical cues, soluble biological signals and metabolic pathways to control various cell behaviors, including proliferation, cell plasticity and stemness essential for tissue regeneration [16]. The shuttling of YAP between cytoplasm and nucleus controls its important role in vascular inflammation [17], such as regulating the phenotype switch of macrophage cells [18]. In inflammation, YAP impairs M2 macrophage polarization and promotes pro-inflammatory cytokine IL6 secretion in the M1 macrophage [18]. However, although the activation of YAP has been extensively studied in other diseases, activating mutations in YAP have not been mentioned in human vascular inflammation. Increasing evidence has indicated that nuclear activation of YAP is accelerated by blood shear stress [19] or implanted materials [20]. This leads to the hypothesis that extracellular stimuli from biophysical factors or materials could be the prime candidates to induce the transcriptional function of YAP.

Here, we provide evidence showing that Mg alloy induces an inflammatory response in human coronary artery endothelial cells (HCAECs). Mg alloy accelerates the accumulation and activation of YAP in the nucleus by suppressing the Hippo pathway. Nuclear YAP further binds to the different DNA regions of inflammatory cytokines, inflammation-related transcription factors and co-transcription factors to initiate their transcription. In addition, nuclear YAP modifies the methylation level of inflammation-related genes through regulating the expression of N6-methyladenosine (m^6^A) modulators (*METTL3, METTL14, FTO* and* WTAP*). Eventually, the expression of inflammation-related genes is increased and inflammation is exacerbated in HCAECs. In contrast, inhibiting YAP represses Mg alloy-induced inflammation. Together, our findings imply that nuclear activation of YAP contributes to Mg alloy-induced inflammation.

## Materials and methods

### Study design

The sham group used in the histologic study represents normal subcutaneous tissue without Mg alloy disc implantation. On the other hand, the control group used in the cellular and molecular biological study indicates that cells are cultured on a dish without contacting Mg alloy disc or the addition of Mg alloy extract medium.

### Cell culture

All cell lines were supplied with 5% CO_2_ at 37°C. HCAECs were obtained from ScienCell^TM^ (San Diego, CA, USA) and cultured in Endothelial Cell Medium (ScienCell^TM^) with 1% fetal bovine serum (ScienCell^TM^), 1% endothelial cell growth supplement (ScienCell^TM^) and 1% penicillin/streptomycin solution (ScienCell^TM^).

### Mg alloy preparation

Mg alloy disc (10 mm × 2 mm) and stent (Ø2 × 18 mm) were obtained from Beijing Amsino Medical Co., Ltd. The component of Mg alloy contains Gd 3.5 ∼ 5.5 wt%, Y 1.5 ∼ 4.5 wt%, Zn 0 ∼ 2.0 wt% and Zr 0 ∼ 2.0 wt%. We polished the metal discs with SiC paper and cleaned them ultrasonically in an acetone bath for 10 min. The Mg alloy extract was prepared using the serum-free Endothelial Cell Medium with the surface area of medium extract ratio of 1.25 cm^2^/mL and incubated at 37°C for 7 days with 5% CO_2_.

### Immersion degradation test

The hydrogen evolution assay was used to monitor the corrosion resistance of the Mg alloy samples in PBS solution and artificial plasma (composition: 6.8 g/L NaCl, 0.4 g/L KCl, 2.2 g/L NaHCO_3_, 0.1 g/L MgSO_4_, 0.2 g/L CaCl_2_, 0.126 g/L Na_2_HPO_4_, 0.026 g/L NaH_2_PO_4_ and 1.0 g/L glucose), respectively. Meanwhile, the pH value of the solution was recorded at the indicated time point. The elements in Mg alloy extract medium after degradation 4 weeks were examined by the inductively coupled plasma mass spectrometry (ICP-MS) (ICAP-QC, Thermo, USA). The detailed process is described previously [13].

### Stent implantation

The New Zealand rabbits (male, 3.0–3.5 kg) are purchased from (DaShuo Experimental Animal Co. Ltd, Chengdu, China). The animal project was approved by the Medical Ethics Committee of Sichuan University. Each stent was hand-crimped on a 3.0 mm angioplasty balloon and intervened into the artery from the proximal iliac artery and then unfolded (10-atm balloon inflation for 30 s) in the artery, achieving an approximate balloon to artery ratio of 1.2:1. Ultrasonic imaging (VINNO 6LAB, Vinno, China) was used to validate the proper location of the stent. The rabbits were given 40 mg aspirin orally 1 day before surgery and daily after that. Euthanasia was performed at 7 days, 14 days and 28 days (*n* = 3 in every group) after stent deployment. HE staining was used to determine the cellular response to stent implantation. Commercial 316 L stainless steel stent was set as the control group for the rabbit stent implantation.

### Subcutaneous implantation

The subcutaneous implantation was used to detect the *in vivo* biodegradation and inflammation-inducing effect of Mg alloy. The male rats are obtained from DaShuo experimental animal Co. Ltd, Chengdu, China, and approved by the Medical Ethics Committee of Sichuan University. The animals were kept at a constant temperature (21 ± 1°C) under 12/12-h light/dark cycle and had free access to water and standard chow. Freshly prepared Mg alloy discs were implanted into individual dorsal subcutaneous pockets. At the indicated time point (7, 14, and 28 days) after implantation, animals were euthanized, and the surrounding tissue was explanted and analyzed to evaluate the inflammation reaction.

### Immunohistochemical staining

Tissues surrounding the Mg alloy disc implanted subcutaneously in rats were fixed in 4% paraformaldehyde overnight and embedded in paraffin blocks according to the well-known protocol. Following the manufacturer’s instructions, blocking and chromogenic detection were performed using the DAKO Envision System with DAB substrate (DAKO Corporation). The detailed protocol is described in Yu *et al.* [21]. The used antibodies are listed in Supplementary Table S2.

### Plasmid construction, lentiviral production and transfection

Briefly, oligonucleotides were cloned into psi-LVRU6GP with the BamHI/EcoRI sites. The sequences of the oligonucleotides are as follows: YAP-sense, 5′-TAATACGACTCACTATAGGG-3′; YAP-antisense, 5′-CTGGAATAGCTCAGAGGC-3′. We designed four YAP1 shRNAs target sequences, and the target sequences are listed in Supplementary Table S3. Plasmids and YAP stably silenced HCAECs were obtained according to the methods described previously [21]. Cells were screened by Endothelial Cell Medium containing 0.5 μg/mL puromycin.

### RNA-sequence

The next-generation sequencing process was performed by Aksomics, Inc (Shanghai, China) and was described previously [21]. Briefly, total RNA was harvested using Trizol reagent (Invitrogen, CA, USA) following the manufacturer’s instructions. After quality control and quantification, total RNA was used to prepare the sequencing library. Then, sequences were performed by Illumina Hiseq 4000 (Illumina, San Diego, CA, USA). *R* package Ballgown was used to choose the differentially expressed genes and transcripts. The correlation analysis was based on gene expression levels (Principal Component Analysis). Only genes with a fold change of ≥1.5 were considered in the subsequent analysis of functional genes.

### Chromatin immunoprecipitation followed sequencing

Chromatin immunoprecipitation (ChIP) was performed as described in Schmidt *et al.* [22]. Antibodies are listed in Supplementary Table S2. Briefly, HCAECs seeding on Mg alloy discs after 24 h was collected. Cells were then fixed with 1% formaldehyde (Thermo Fisher Scientific, Waltham, MA, USA) in the medium for 10 min at room temperature and lysed in the cell lysis solution. The chromatin fragments were sheared to ∼200 bp by using the ultrasonic processor (Thermo Fisher Scientific, Waltham, MA, USA). Anti-YAP antibody was incubated with the sheared chromatin overnight at 4°C. Antibody/antigen complexes were recovered with ProteinA-Dynabeads (Thermo Fisher Scientific, Waltham, MA, USA) for 2 h at 4°C. The sequencing of ChIP’d DNA was performed by Aksomics, Inc (Shanghai, China) and was described in Guo *et al*. [23].

### ChIP-seq data analysis

After the sequencing, the data from the sequencing platform were analyzed. Off-Line Basecaller software (OLB V1.8) was used to perform base-calling. FastQC software was used to check the sequence quality. After passing Solexa CHASTITY quality filter, the clean reads were aligned to the human reference genome UCSC HG19 using BOWTIE (V2.1.0). Aligned reads were used for peak calling of the ChIP regions using MACS V1.4.2. Statistically significant ChIP-enriched regions (peaks) were identified by comparison of IP vs Input or comparison to a Poisson background model, using a *P* value threshold of 10^−4^. Then the peaks were annotated by the nearest gene using the newest UCSC RefSeq database.

### ChIP-PCR

ChIP-qPCR was performed as described previously [21]. Briefly, per 5 μg antibody was used to target per 100 μg sheared chromatin. Quantitative real-time PCR (qRT–PCR) was conducted with a Rotor-Gene Q (Qiagen) thermal cycler. Each sample was analyzed by three independent experiments.

### ChIP-qPCR data analysis

Normalizing the Ct value of each ChIP DNA fraction to the Input DNA fraction Ct value for the same qPCR Assay (ΔCt) was used to represent chromatin sample preparation differences. The % Input of each ChIP fraction was calculated by the following formula: %Input = 2 ^(CtInput—CtChIP)^ × Fd × 100%. Fd means the Input dilution factor. Here, Fd is 1/5. Fold Enrichment = [%(ChIP/Input)]/[% (Negative control/Input)]. Adjust the normalized ChIP fraction Ct value for the normalized background (mock IP) fraction Ct value. ΔΔCt [ChIP/mock IP] = ΔCt [normalized ChIP] – ΔCt [normalized mock IP]. PCR primers are listed in Supplementary Table S4.

### Motif discovery in ChIP-seq peaks

YAP peaks motif is discovered by the DREME software online (4.12.0) [24].

### Methylation microarray

The methylation microarray was performed by Aksomics, Inc (Shanghai, China) and was described previously [21].

### Microarray data analysis

The microarray data analysis was carried out according to the methods described previously [21].

### Cell proliferation assay

HCAECs were seeded in a 96-well plate (1000 cells/well) and incubated at 37°C with supplementation of 5% CO_2_. The cells were cultured 24 h before conducting MTT(3-[4,5-dimethylthiazol-2-yl]-2,5 diphenyl tetrazolium bromide) assay. The MTT assay (Sigma-Aldrich, USA) was carried out following the manufacturer’s instructions; 570 nm absorbance was measured.

### Western blotting

Western blotting was conducted as described previously [25]. All experiments were quantified by Image lab (Bio-Rad) and normalized to internal control. All antibodies used in this study are provided in Supplementary Table S2.

### Immunofluorescence

After culturing in Mg alloy extract, cell samples were washed twice with ice-cold PBS and added suitable 4% paraformaldehyde for 30 min at room temperature, then washed three times with PBS. Cells were permeabilized and blocked in PBS containing 5% goat serum and 0.1% Triton X-100 for 30 min at room temperature. Primary antibodies were used to incubate the cells overnight at 4°C and secondary antibody in PBS containing 5% goat serum were incubated for 1 h at room temperature. The nucleus was stained with DAPI for 10 min at room temperature. The location and expression of the aim protein were observed from three randomly selected views. All antibodies used in this study are listed in Supplementary Table S2.

### RNA extraction and qRT–PCR

To determine the mRNA expression level of target proteins, we harvested total RNA from cells with Trizol reagent (Invitrogen, CA, USA) following the manufacturer’s protocol. cDNA was synthesized by reverse transcription using RevertAid First Strand cDNA Synthesis Kit (Thermo Fisher Scientific, Waltham, MA, USA). Amplification reactions were performed in 20 mL reactions using CFX96TM Real-Time PCR Detection System. Each sample was analyzed in triplicate. The cycle thresholds (Ct) were determined using CFX Manager 3.1 software, and GAPDH or β-actin was used as the internal control. PCR primers are listed as Supplementary Table S4.

### Statistical analysis

Statistical analyses were conducted using GraphPad Prism v5.02 for Windows (GraphPad Software, San Diego, CA, USA). One-way analysis of variance (ANOVA) followed by Tukey’s test or two-tailed unpaired *t*-test was used to detect the statistical significance. Statistical significance of ChIP-PCR is performed by two-way ANOVA. In this study, all biochemical experiments were conducted at least three times, and the representative images were shown. Results represent mean ± SD.

### Availability of data

Gene expression profile data and ChIP-seq data have been deposited for public access in the NCBI Gene Expression Omnibus under Accession Number (GSE146167, GSE146168). The methylation microarray data are deposited under GSE146038. All data supporting the findings of this study are displayed in the paper and supplementary materials. It is available from the corresponding author upon reasonable request.

## Results

### The degradation behavior of Mg alloy *in vitro* and *in vivo*

**Figure 1.**
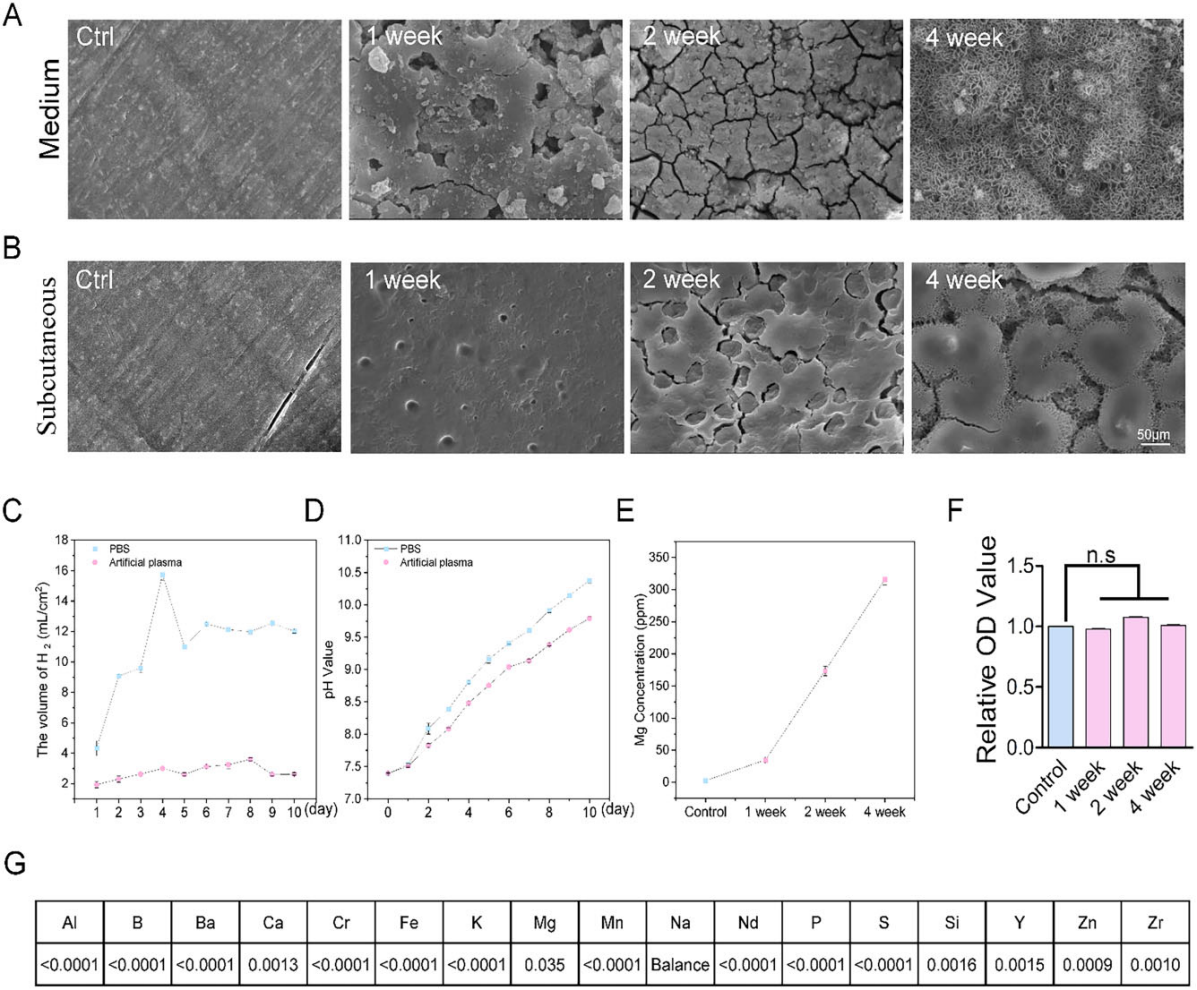
The degradation behavior of Mg alloy *in vitro* and *in vivo*. (**A** and **B**) The scanning electron microscope (SEM) images showed the surface morphologies of degraded discs in medium and subcutaneous tissue at different time duration, scale bar, 50 μm. (**C**) The volume of H_2_ evolutions of Mg alloy disc in PBS and artificial plasma for every 1 day degradation. (**D**) pH values shifted during the Mg alloy degradation process in PBS and artificial plasma. (**E**) The Mg^2+^ concentration curve in the degradation duration. (**F**) The viability of HAECs at indicated degradation duration was checked by MTT assay. (**G**) The released elements in Mg alloy extract medium after 4 weeks of degradation were examined by the ICP-MS; the values mean the percentage of mass/volume ratio. Data are expressed as mean ± SD, *P-*values were calculated according to one-way analysis of variance followed by Tukey’s test, n.s denotes not significant

First, we measured the mechanical properties of the Mg alloy materials (the components of Mg alloy are described in Material and methods), shown in Table 1. The degradation behavior of the Mg alloy was evaluated *in vitro* and *in vivo*. The results implied that the *in vivo* corrosion of an Mg alloy is relatively slower than *in vitro* corrosion (Fig. 1A and B), also in agreement with previously published results [26]. Furthermore, the H_2_ evolution volume (Fig. 1C) generated in artificial plasma is less than in PBS solution during the investigated period. It is in line with the pH shift in the degradation process (Fig. 1D). The elevated Mg^2+^ concentration was positively correlated with the degradation duration (Fig. 1E). We further detected the released elements in the Mg alloy extract medium (Fig. 1G). The effect of Mg alloy extract medium at indicated degradation duration on cell viability was detected by MTT assay. A significant difference was not observed, suggesting the Mg alloy used in this study has good biocompatibility (Fig. 1F). 

**Table 1.** Mechanical properties of the magnesium alloy materials used in this study

Samples	Yield strength (Rp_0.2_)	Tensile strength (Rm)	Elongation (A)
Polylactic acid (PLA)	35 MPa	60 MPa	3.8%
Stainless steel (316 L)	170 MPa	480 MPa	45%
Mg alloy	189.44 MPa	264.21 MPa	22.34%

### Mg alloy induces an inflammatory reaction in HCAECs

**Figure 2.**
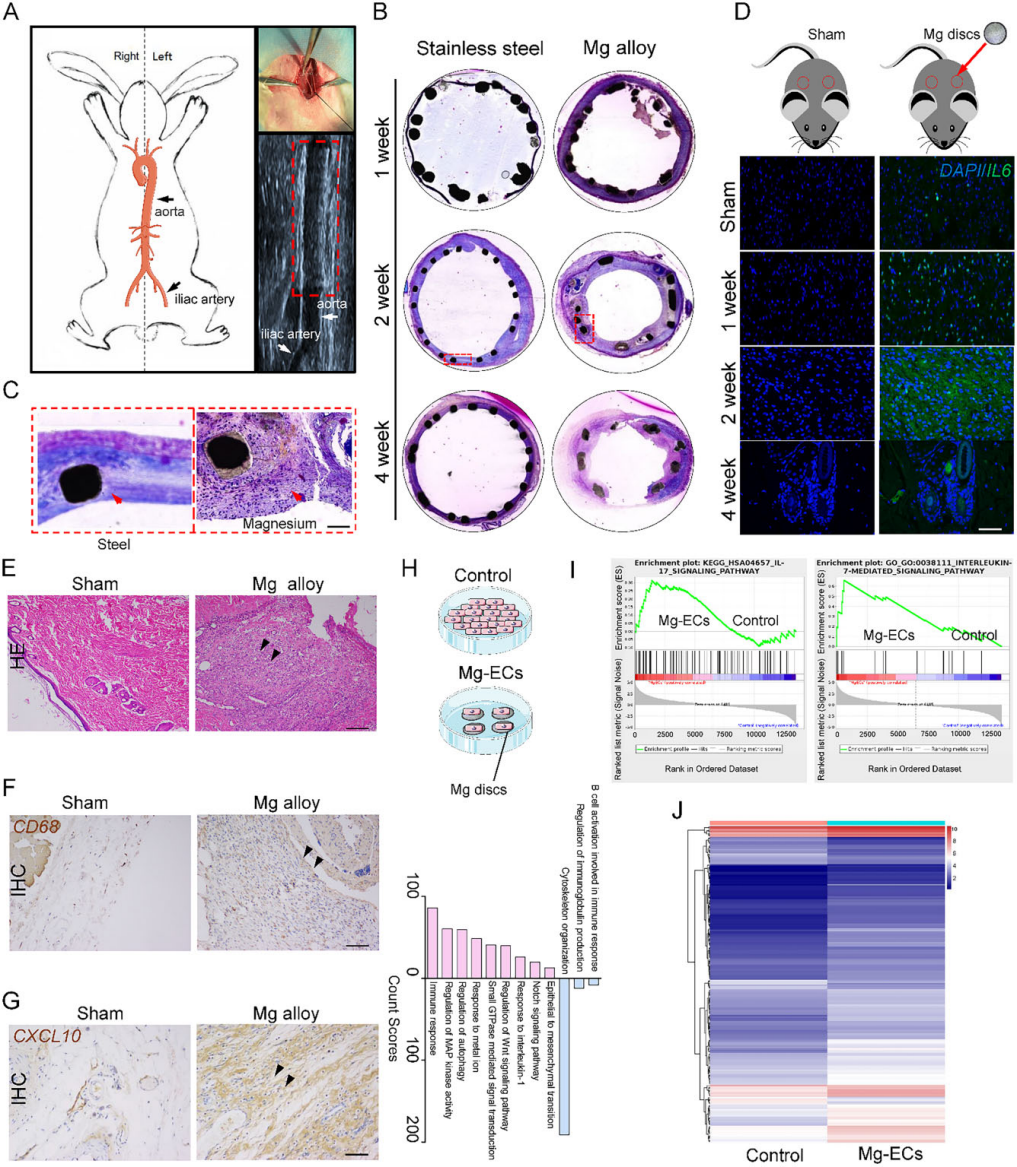
Mg alloy induces inflammation in HCAECs. (**A**) The schematic diagram shows the stent implantation through the rabbit iliac artery. (**B** and **C**) HE staining shows the accumulation of inflammatory cells on Mg alloy stent, scale bar, 50 μm. (**D**) Immunostaining illustrates the increased expression of IL6 in the tissue around Mg alloy disc subcutaneous implantation, scale bar, 50 μm. (**E**) HE staining of subcutaneous tissue from rat with Mg alloy disc implantation, scale bar, 50 μm. (**F** and **G**) Immunohistochemical staining of CD68 and CXCL10, a marker of I-type macrophage, from subcutaneous tissue with Mg alloy disc implantation, scale bar, 50 μm. (**H**) Biologic process (BP) analysis for the Mg-ECs (up-regulated colored red, down-regulated colored blue). (**I**) GSEA analysis of pro-inflammatory genes in Mg-ECs. (**J**) The differently expressed genes involved in inflammation from RNA-seq data

We then evaluated the inflammatory response in vessels with Mg alloy stent implantation; the process of stent implantation is shown in Fig. 2A. HE staining revealed that the Mg alloy stent induced the accumulation of inflammatory cells (Fig. 2B and C). To further confirm the pro-inflammatory effect of Mg alloy, Mg alloy disc was implanted subcutaneously. Immunostaining showed increased expression of pro-inflammatory cytokine IL6 in the tissue around the Mg alloy discs compared with the sham group (Fig. 2D). In line with this, macrophage cells accumulated, which is associated with the higher expression of the M1 macrophage marker CXCL10 after 2 weeks implantation [27] (Fig. 2E–G). Collectively, the above data suggested that Mg alloy induced an inflammatory response after implantation.

However, whether Mg alloy stent directly initiates inflammation in endothelial cells remains unknown. Therefore, we conducted global gene expression profiling of HCAECs cultured on Mg alloy discs for 24 h (set as Mg-ECs, HCAECs cultured on the dish are referred to as Control). GO (Gene Ontology) functional annotation indicated that Mg alloy activated inflammatory biological processes (Fig. 2H). Gene set enrichment analysis revealed that IL17 inducing IL6 secretion [28], increased dramatically. Meanwhile, elevated IL7, which stimulated lymphoid progenitor proliferation [29], was observed (Fig. 2I). The mRNA-seq data in the heatmap displayed the differently expressed genes (*P* < 0.05 and absolute fold change ≥1.5) involved in inflammatory response (Fig. 2J, the detailed genes are listed in Supplementary Table S1). The up-regulation of inflammatory genes in heatmap was verified by quantitative real-time PCR (qRT–PCR) analysis (Supplementary Fig. S1B–I). In addition, the inflammatory reaction was also found in HCAECs and tissues after exposure to AZ-31, a different Mg alloy, which was used predominantly as the control material in the Mg alloy study (Supplementary Fig. S2).

### Mg alloy is a material stimulus for Yap activation in HCAECs

**Figure 3.**
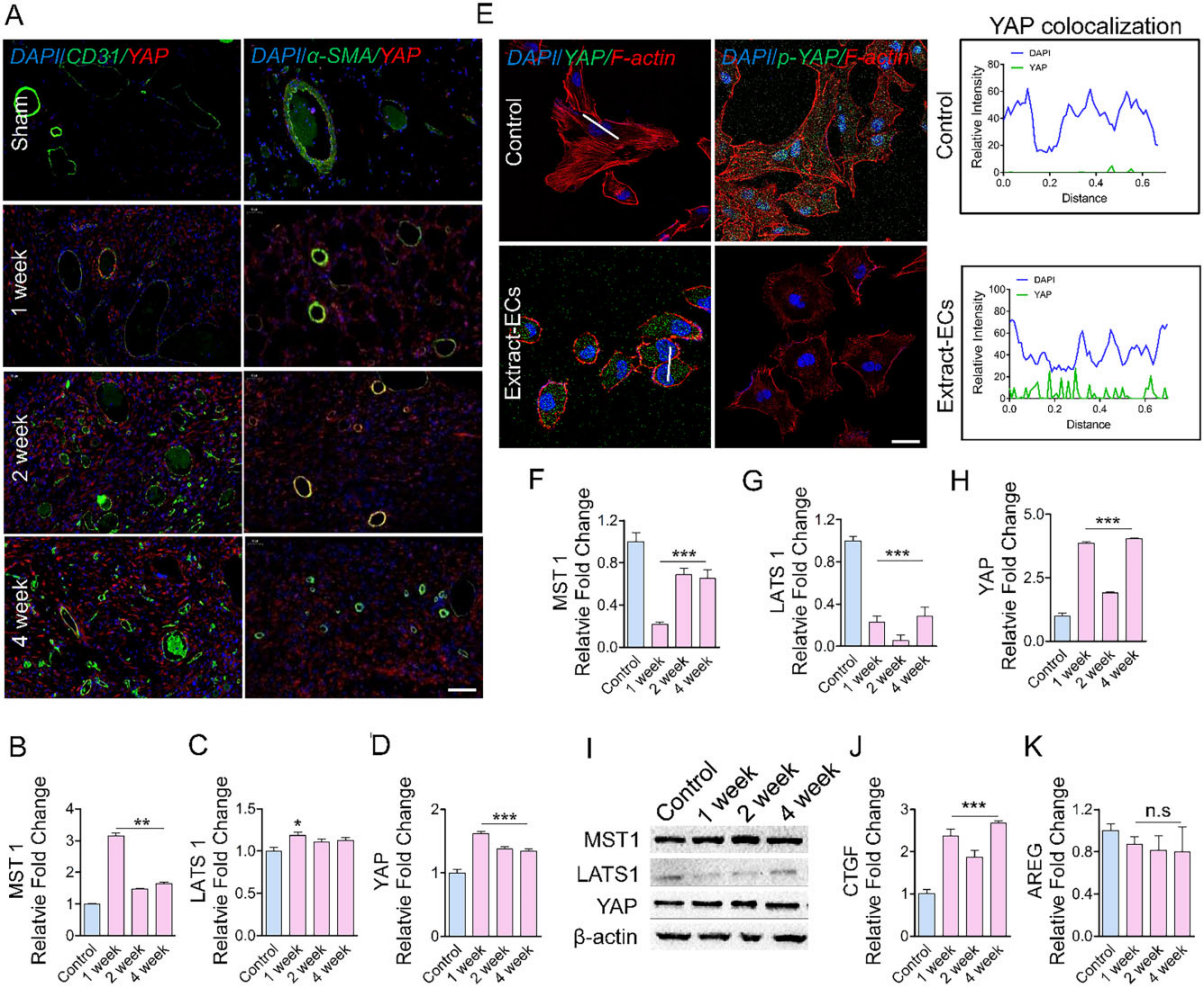
Mg Alloy induces Yap activation in HCAECs. (**A**) Immunostaining shows the expression and location of Yap in vascular cells around the subcutaneous Mg alloy discs, CD31: the marker of endothelial cells; α-SMA: smooth muscle cells; scale bar, 50 μm. (**B–D**) Expression of Yap upstream genes *MST1* and *LATS1* in the tissue around Mg alloy discs was measured by qRT–PCR analysis (*n* = 3). (**E**) Immunostaining was used to check the Yap and phosphorylated Yap in Extract-ECs; the white line indicates the statistical co-location area of Yap, scale bar, 10 μm. (**F–I**) Expression of Yap, MST1 and LATS1 at the protein and gene level in HCAECs were validated by Western blot and qRT–PCR analysis (*n* = 3). (**J** and **K**) the well-recognized Yap target genes (*CTGF, AREG*) were measured by qRT–PCR analysis (*n* = 3). Data are presented as mean ± SD, *P-*values were calculated according to one-way analysis of variance followed by Tukey’s test, n.s means not significant, *P < 0.05, **P < 0.01, ***P < 0.001

We then investigated the potential molecular mechanisms of Mg alloy initiating inflammation. As a downstream factor of the Hippo pathway, YAP is reported to contribute to inflammation in blood vessels [19]. The overproduction of YAP was found in endothelial and smooth muscle cells, the fundamental cell components of vessels, around the Mg alloy discs (Fig. 3A). The inactivation of MST1 and LATS1 comprised Hippo pathways that were responsible for YAP activation [30]. The increased expression of YAP in the tissues around Mg alloy was found, while the expression of MST1 and LATS1 were elevated as well. This may be caused by the heterogeneity of tissues (Fig. 3B–D).

The transcriptional activity of phosphorylated YAP was inhibited via cytoplasmic retention and degradation [31]. To further confirm that Mg alloy activates YAP during the degradation process, we measured the phosphorylation level and location of YAP in HCAECs with subjection to the Mg alloy extract medium. We found that Mg alloy extract medium repressed the phosphorylation of YAP and promoted it into the nucleus of HCAECs (Fig. 3E), accompanied by reduced Hippo kinases and increased YAP (Fig. 3F–H). The phenomenon at the gene level was further verified by western blot analysis. Consistently, LATS1 was reduced, and YAP had a higher expression (Fig. 3I). The well-characterized YAP target gene *CTGF* was also up-regulated (Fig. 3J and K).

### Yap has a transcriptional response to Mg alloy

**Figure 4.**
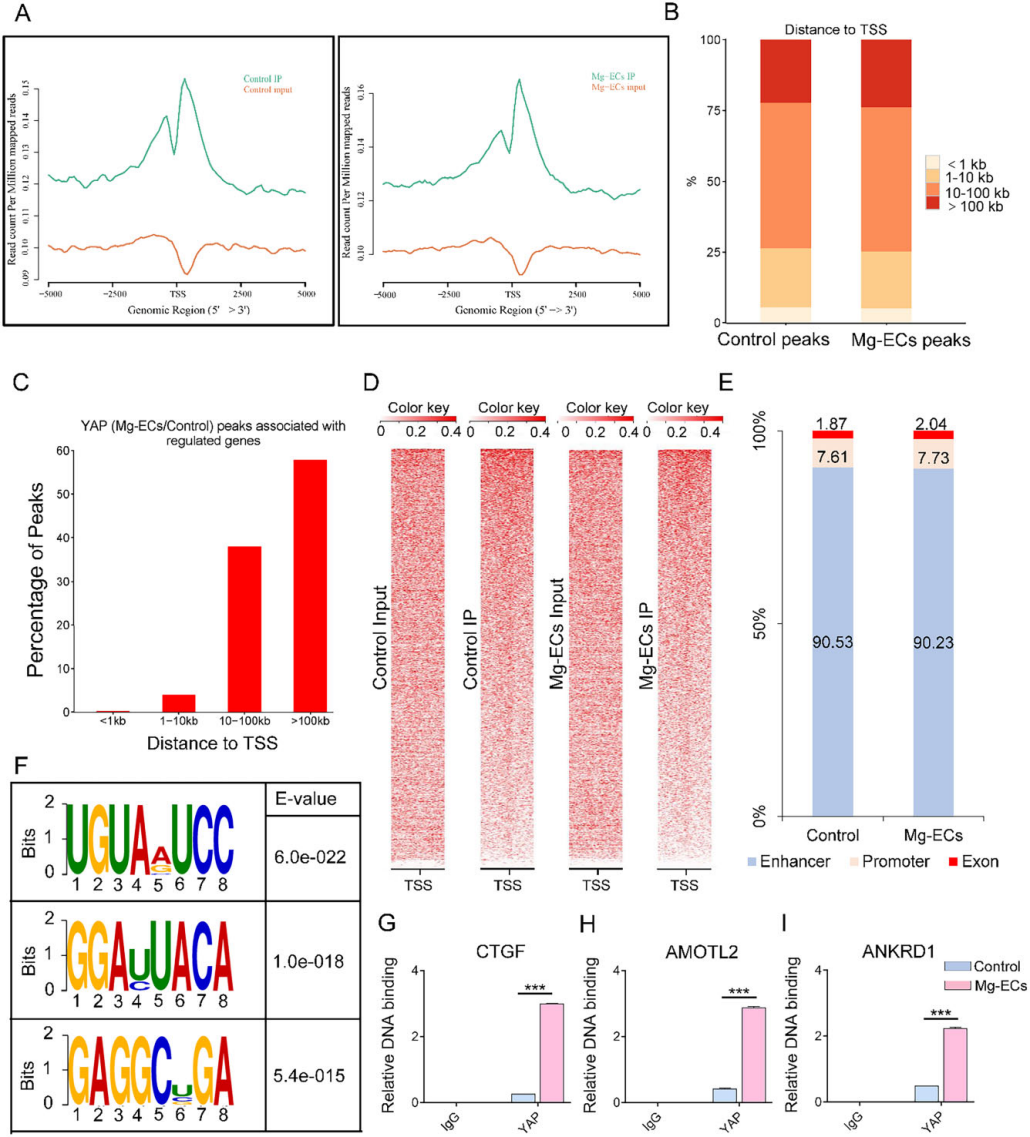
Yap shows transcriptional function upon Mg alloy stimuli. (**A**) Distribution of peak regions around transcription start sites (TSS, ±5 kb to TSS), the distance to TSS is labeled on the X-axis, and Y-axis displayed read count per million mapped reads. (**B**) Absolute distance of Yap peaks to the nearest TSS from. (**C**) Distance between Yap-binding sites and the TSS of the direct target genes they are associated to. (**D**) Density maps of Yap ChIP-seq reads at Yap bound loci. (**E**) Fraction of Yap peaks associated with each category. (**F**) The sequence logo shows the top motifs enriched across altered Yap peaks identified from Mg-ECs. (**G–I**) ChIP-PCR analysis validated the genes where Yap binds to. Data are shown as mean ± SD, *P-*values were calculated according to two-way analysis of variance, ***P < 0.001

To elucidate how YAP contributes to the Mg alloy-induced inflammation, we conducted ChIP assays with YAP antibodies followed by next-generation sequencing (ChIP-seq). The position of YAP peak summits located near the transcription start site (TSS) revealed the transcriptional activator function of YAP (Fig. 4A). Furthermore, we analyzed the distribution of YAP-binding sites relative to genes annotated in the human genome. Only a few peaks mapped close (≤1 kilobase (kb)) to TSS was found, whereas most peaks were located farther than 10 kb from the closest TSS (Fig. 4B and C). Heatmap represented the location of YAP-binding sites (Fig. 4D). Here, according to their distances to TSS and the functional characters, we grouped the peaks into three classes (enhancer, promoter and exon). Notably, only a very small fraction (7–8%) of YAP peaks were located on promoters; instead, over 90% of peaks were located in enhancer regions (Fig. 4E). In addition, the YAP peaks were characterized by the canonical UGUAVUCC motif (Fig. 4F). To ensure the accuracy of identifying YAP-binding sites from the ChIP-seq data, we conducted ChIP-PCR to validate the relative DNA binding of several well-characterized YAP target genes, such as *CTGF, AMOTL2* and *ANKRD1* [15]. The relative DNA binding of *CTGF, AMOTL2* and *ANKRD1* increased in Mg-ECs (Fig. 4G–I).

### Yap modulates transcription of inflammation-related genes

**Figure 5.**
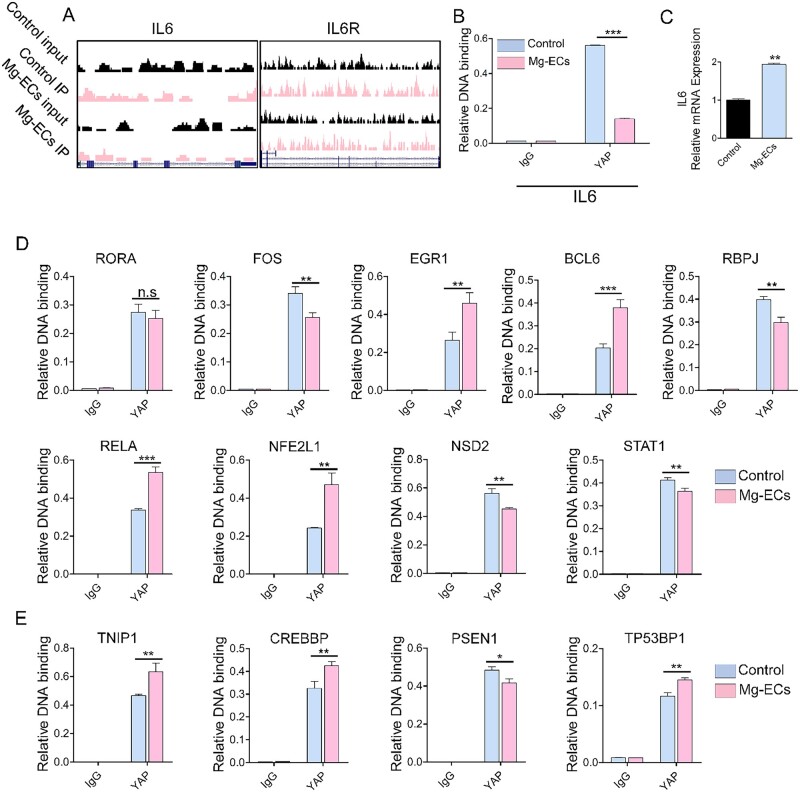
Yap regulates inflammation-related genes transcriptionally. (**A**) IL6 and IL6R peaks where Yap binds to the promoter or enhancer, respectively. (**B**) Validation by ChIP-qPCR analysis of Yap binding to *IL6* in Mg-ECs. (**C**) qRT–PCR analysis of *IL6* in Mg-ECs. (**D**) ChIP-qPCR analysis of Yap binding to the promoter of inflammation-related transcriptional factors. (**E**) ChIP-qPCR analysis of Yap binding to the promoter of inflammation-related co-transcriptional factors. Data are presented as mean ± SD, *P-*values were calculated according to a two-tailed unpaired *t*-test or two-way analysis of variance. **P < 0.05, **P < 0.01, ^***^P < 0.001

Subsequently, we sought further to reveal the transcriptional role of YAP on inflammation-related genes. YAP peaks were located at the promoter and enhancer region of IL6 (Fig. 5A) and the decreased relative DNA binding of *IL6* (Fig. 5B) led to increased mRNA expression (Fig. 5C). Besides binding with the DNA functional region of inflammatory cytokines to regulate their mRNA expression directly, YAP could also bind to the promoter region of inflammation transcriptional factors (Fig. 5D) and co-transcriptional factors (Fig. 5E) and thus regulate their mRNA expression (Supplementary Fig. S3C–O). Moreover, except for promoter and enchanter elements, we found that YAP binds to other DNA functional regions, such as exons, to regulate the expression of inflammation-related genes (Supplementary Fig. S4, the definition of inflammation-related genes came from UniProt database).

### Yap modifies the mRNA methylation of inflammation-related genes

**Figure 6.**
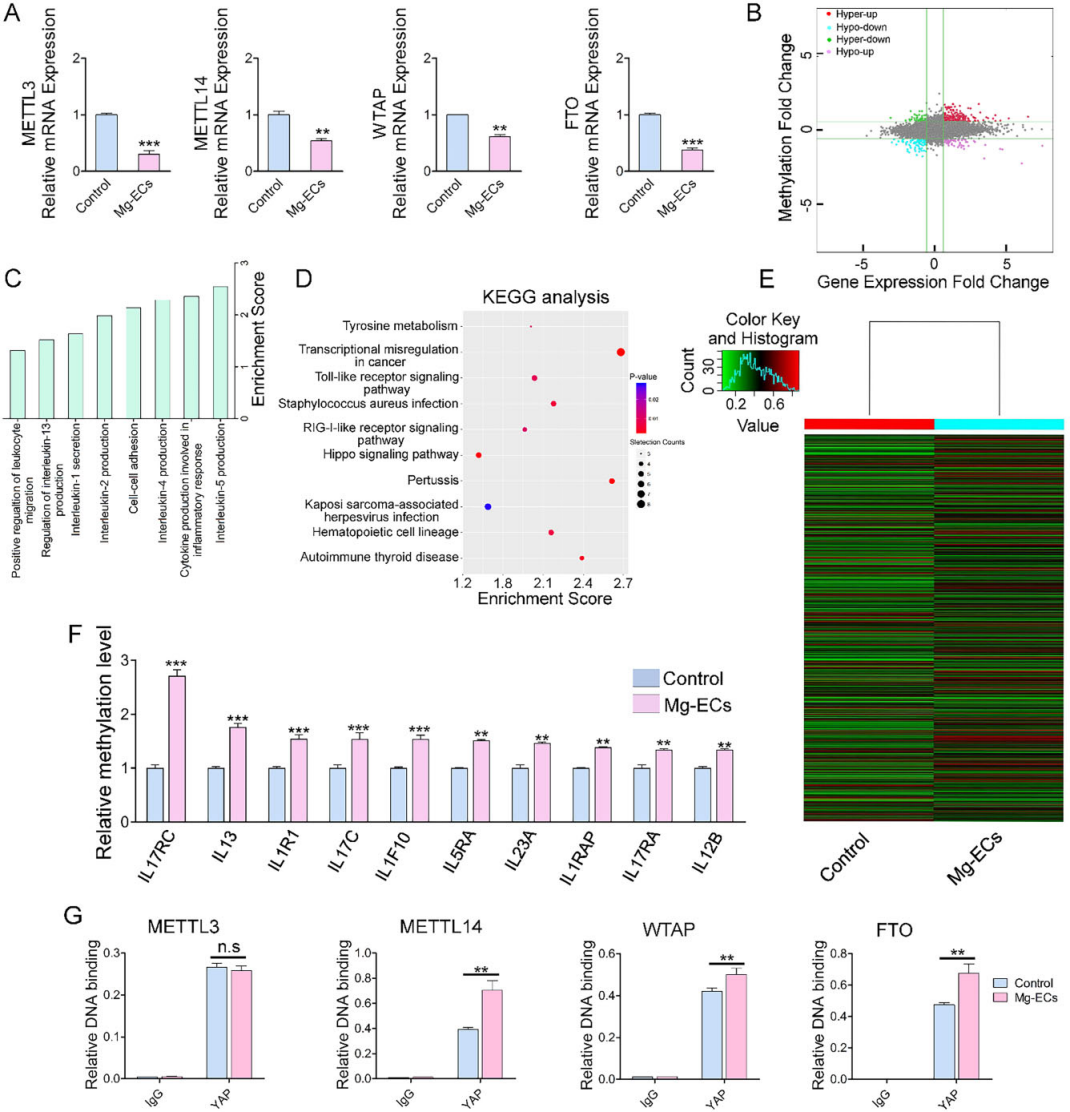
Yap modulates the methylation of inflammatory genes. (**A**) The mRNA expression of *METTL3*, *METTL14*, *WTAP* and *FTO* were examined by qRT–PCR (*n* = 3). (**B**) The distribution of transcript with a different regulation in both m^6^A level and expression in Mg-ECs. (**C**) Major enriched and meaningful biological process terms of m^6^A peaks transcripts. (**D**) Significantly enriched pathways of m^6^A peaks transcripts. (**E**) Hierarchical clustering analysis showing the differentially methylated mRNAs. (**F**) The methylation level analysis of inflammation-related genes from m^6^A-mRNA microarray data. (**G**) ChIP-qPCR analysis of Yap binding with methylation modulators. Data are presented as mean ± SD, *P-*values were calculated according to two-tailed unpaired *t*-test or two-way analysis of variance, n.s denotes not significant,**P < 0.01, ***P < 0.001

Epitranscriptomic changes of RNA may post-transcriptionally regulate gene expression. RNA methylation to form m^6^A in mRNA is the most prevalent mRNA internal modification and is regarded as a universal regulatory mechanism of gene expression [32]. *METTL3, METTL14, WTAP* and *FTO*, which are responsible for m^6^A methylation [33], decreased significantly in Mg-ECs (Fig. 6A). Next, a methylation microarray was conducted to validate the different methylation levels in Mg-ECs. Microarray profiling showed that 193 transcripts were significantly hyper-methylated with increased mRNA expression, 58 transcripts were significantly hyper-methylated with decreased mRNA expression, 89 transcripts were significantly hypo-methylated with decreased mRNA expression and 58 were significantly hypo-methylated with increased mRNA expression (Fig. 6B). The profile indicated that Mg alloy induced a significantly changed methylation level in HCAECs. Gene ontology analysis and KEGG pathway analysis suggested that different methylation was associated with the inflammation response and Hippo pathway, which was the key regulator of YAP phosphorylation and activation (Fig. 6C and D). Hierarchical clustering revealed distinguishable gene expression patterns between ECs and Mg-ECs (Fig. 6E). Inflammation-related genes had an obvious increased methylation level (Fig. 6F). Finally, ChIP-PCR results provided direct evidence that the relative DNA binding of YAP with methylation regulators had a significant increase in expression except for *METTL3* (Fig. 6G). This suggested that YAP modulated the mRNA expression of methylation regulators through its transcriptional function. Accordingly, YAP was responsible for the varied methylation induced by Mg alloy.

### Yap inhibition retards the Mg-alloy induced inflammation

**Figure 7.**
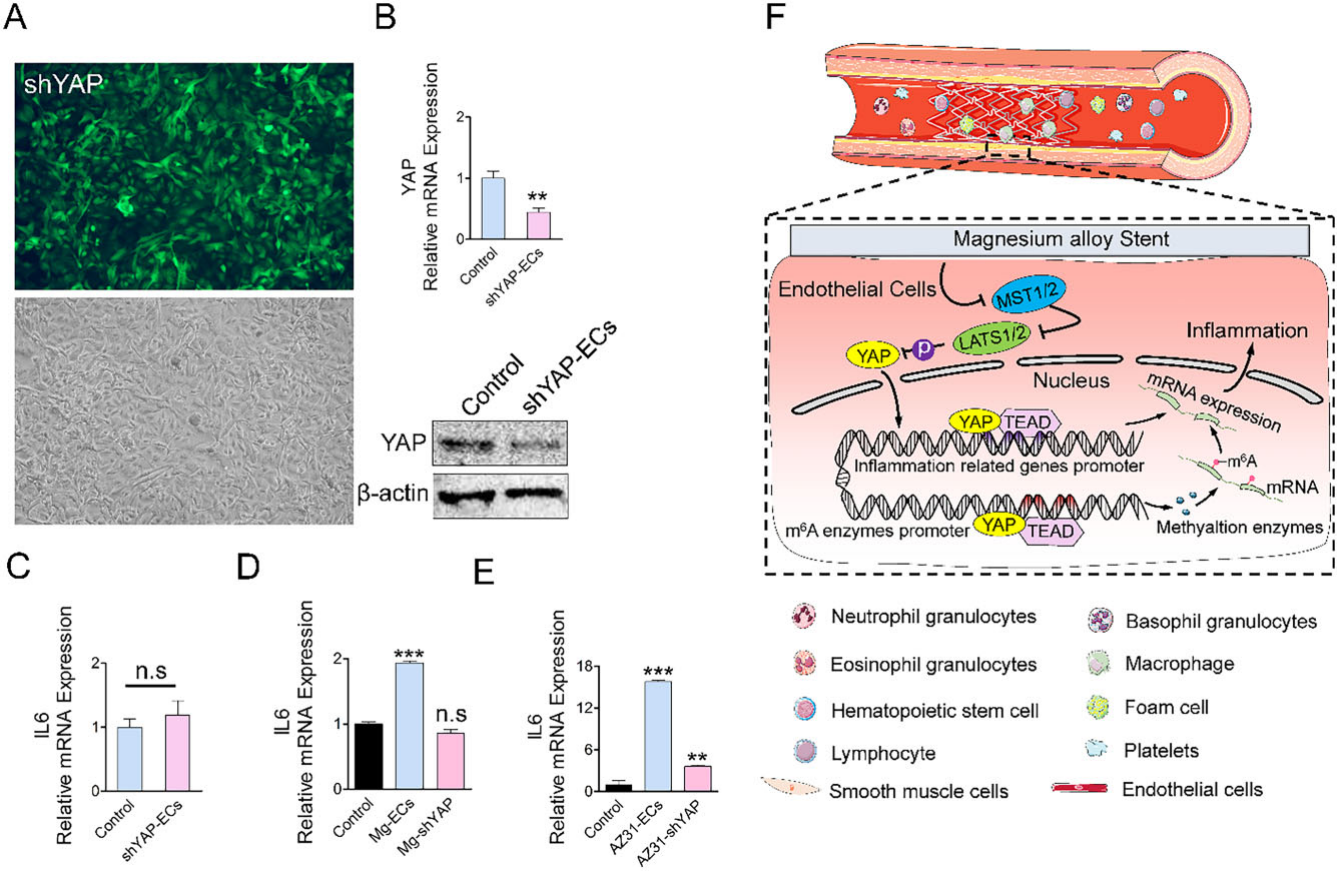
Activation of Yap contributes to Mg alloy-induced inflammation. (**A** and **B**) The transfection efficiency of HCAEC transfected with shRNA against Yap was validated. (**C** and **D**) qRT–PCR analysis of *IL6 and IL10* in shYAP-ECs. (**E–H**) qRT–PCR analysis of *IL6 and IL10* in HCAECs cultured in different magnesium alloy extract medium. (**F**) Inflammation induces the accumulation of inflammatory cells, such as macrophages and lymphocytes. Mg alloy initiates inflammation in HCAECs by suppressing hippo pathway and, in turn, causes Yap nuclear shuttling, which regulates the transcription and methylation of inflammation-related genes to modulate their mRNA expression. Data are presented as mean ± SD, *P-*values were calculated according to two-tailed unpaired *t*-test or one-way analysis of variance followed by Tukey’s test, n.s denotes not significant, **P < 0.01, ***P < 0.001

To further validate the role of YAP in Mg alloy-induced inflammation, we knocked down YAP in HCAECs by an shRNA approach (referred to as shYAP-ECs, the transfection efficacy was shown in Fig. 7A and B) and measured the expression of the inflammatory gene. In shYAP-ECs, *IL6* had a stable gene expression (Fig. 7C). Most importantly, shYAP-ECs cultured on two kinds of Mg alloys did not display elevated *IL6* (Fig. 7D and E)*.* These results provided strong evidence suggesting that Mg alloy*-*induced inflammation was dependent on the nuclear activation of YAP.

In conclusion, we demonstrated that Mg alloy stent initiated the inflammatory response in HCAECs, and this inflammatory response resulted in inferior migration and adhesion ability of HCAECs. Meanwhile, Mg alloy suppressed the Hippo pathway leading to the nuclear accumulation of YAP. The activated YAP further regulated transcription and methylated modification of inflammation-related genes, which together promoted their expression (Fig. 7F).

## Discussion

Currently, the rates of definite stent thrombosis in bare-metal stent (BMS) and drug-eluting stents (DES) are estimated to be < 2% [34]. However, for either BMS or DES, the metallic stent is designed to be kept in the vessel for a long period. When the stented vessel has restenosis, it becomes difficult to deliver a new stent to the injured vessel due to the occupancy of the previous stent. In addition, permanent vessel caging impairs arterial physiology and increases the probability of very late stent thrombosis [35]. Therefore, a degradable stent has recently attracted attention. After the vessel has healed sufficiently, a degradable stent would disappear.

Compared with polymeric materials, degradable metallic materials have a superior combination of strength and ductility. Therefore, they have gained more attention in vascular stent application. Most commonly, degradable metallic stents have been made from Mg alloy. However, the clinical applications of Mg alloy are limited mainly by their rapid degradation rates and local tissue inflammation [36]. Extensive efforts *in vitro* focus on how to retard the corrosion rate of Mg alloy, and our previous work showed that bionic tea stain-like nanoparticle coatings and modification of components in Mg alloy were able to protect from corrosion [37, 38]. Although those improvements of Mg alloy inhibit the corrosion rate compared with bulk material, the inflammation accompanied by the accumulation of inflammatory cells, such as macrophages, still occurs [12].

Increasing evidence suggested that some novel Mg alloy and surface coatings could downregulate inflammatory response by regulating the phenotypic switching of macrophages after implantation [11–13]. On the other hand, rapid coverage of endothelial cells on stents could provide mechanical support for degrading Mg alloy and encase the degraded residue, thus avoiding a drastic inflammatory reaction. For that reason, figuring out whether and how Mg alloy induces inflammation in endothelial cells should be considered. Here, we showed that in the early implantation, the inflammatory response was caused by Mg alloy. Inflammatory cytokines IL6 increased dramatically (Fig. 2D). Gene profile analysis illustrated that Mg alloy modified the biological process and significantly changed genes related to inflammation in HCAECs (Fig. 2 H–J). It suggested that Mg alloy caused an inflammatory response in HCAECs. It should be noted that the Mg alloy used in this study lacks any coating, and the components are unique; therefore, the pro-inflammatory response is a specific phenomenon for the Mg alloy used in this study.

YAP was known to translocate into the nucleus and initiate transcription upon extracellular stimuli and likely contributes to inflammation-related diseases [19, 39]. Here, we found that the Mg alloy induced the nuclear activation and high expression of YAP in HCAECs (Fig. 3). Therefore, after delivering Mg alloy stent, activated YAP might aggravate the inflammatory reaction in the atherosclerotic vessel. It highlighted the pro-inflammatory role of YAP in inflammation derived from Mg alloy implantation. Moreover, we found that, as a transcription regulator, activated YAP further regulated the expression of inflammation transcription factors and co-transcription factors through direct binding (Fig. 5D and E), which together promoted the transcription of the inflammatory gene. Except for the transcriptional regulation, YAP could regulate the expression of the inflammatory genes at the post-transcriptional level. We demonstrated that YAP bound to m^6^A modulators (*METTL3, METTL14, WTAP* and* FTO*) (Fig. 6G) to regulate their mRNA expression, which changed the methylation level of inflammatory genes. Collectively, activated YAP regulates the transcription and mRNA methylation of inflammatory genes and finally promotes their expression. During the degradation of Mg alloys, various factors could activate YAP, such as the change of the mechanical property of Mg alloy, pH shift and the alloying ions. It is not easy to identify a specific factor activating YAP because the activating function of these factors is synergetic or independent. Therefore, terminating the transcriptional role of YAP might be the potential strategy to inhibit inflammation after Mg alloy implantation.

Increasing evidence has shown that some clinically used drugs may be used to inhibit YAP nuclear allocation and transcriptional responses. Statin was shown to inhibit the SREBP (Sterol Regulatory Element-Binding Protein)/mevalonate pathway to suppress the YAP nuclear activation [40]. In addition, an engineered peptide was designed to interrupt the YAP–TEAD interaction to inhibit the transcriptional function of YAP [41, 42]. In this study, we demonstrated that Mg alloy-induced inflammation was dependent on the nuclear translocation and transcriptional function of YAP. Therefore, statins and engineered peptides have potent anti-inflammatory properties, and YAP suppression may be a new therapy that alleviates inflammation in the application of Mg alloy coronary stent.

## Supplementary Material

rbac002_Supplementary_Data

## References

[1] Benjamin EJ , Muntner P, Alonso A, Bittencourt MS, Callaway CW, Carson AP, Chamberlain AM, Chang AR, Cheng S, Das SR, Delling FN, Djousse L, Elkind MSV, Ferguson JF, Fornage M, Jordan LC, Khan SS, Kissela BM, Knutson KL, Kwan TW, Lackland DT, Lewis TT, Lichtman JH, Longenecker CT, Loop MS, Lutsey PL, Martin SS, Matsushita K, Moran AE, Mussolino ME, O'Flaherty M, Pandey A, Perak AM, Rosamond WD, Roth GA, Sampson UKA, Satou GM, Schroeder EB, Shah SH, Spartano NL, Stokes A, Tirschwell DL, Tsao CW, Turakhia MP, VanWagner LB, Wilkins JT, Wong SS, Virani SS; American Heart Association Council on Epidemiology and Prevention Statistics Committee and Stroke Statistics Subcommittee. Heart disease and stroke statistics-2019 update: a report from the American Heart Association. Circulation 2019;139:e56–28. 30700139 10.1161/CIR.0000000000000659

[2] Moravej M , Mantovani D. Biodegradable metals for cardiovascular stent application: interests and new opportunities. Int J Mol Sci 2011;12:4250–70. 21845076 10.3390/ijms12074250 PMC3155349

[3] Chen Y , Xu Z, Smith C, Sankar J. Recent advances on the development of magnesium alloys for biodegradable implants. Acta Biomater 2014;10:4561–73. 25034646 10.1016/j.actbio.2014.07.005

[4] Staiger MP , Pietak AM, Huadmai J, Dias G. Magnesium and its alloys as orthopedic biomaterials: a review. Biomaterials 2006;27:1728–34. 16246414 10.1016/j.biomaterials.2005.10.003

[5] Haude M , Erbel R, Erne P, Verheye S, Degen H, Böse D, Vermeersch P, Wijnbergen I, Weissman N, Prati F, Waksman R, Koolen J. Safety and performance of the drug-eluting absorbable metal scaffold (DREAMS) in patients with de-novo coronary lesions: 12 month results of the prospective, multicentre, first-in-man BIOSOLVE-I trial. Lancet 2013;381:836–44. 23332165 10.1016/S0140-6736(12)61765-6

[6] Haude M , Ince H, Abizaid A, Toelg R, Lemos PA, von Birgelen C, Christiansen EH, Wijns W, Neumann F-J, Kaiser C, Eeckhout E, Lim ST, Escaned J, Garcia-Garcia HM, Waksman R. Safety and performance of the second-generation drug-eluting absorbable metal scaffold in patients with de-novo coronary artery lesions (BIOSOLVE-II): 6 month results of a prospective, multicentre, non-randomised, first-in-man trial. Lancet 2016;387:31–9. 26470647 10.1016/S0140-6736(15)00447-X

[7] Toutouzas K , Colombo A, Stefanadis C. Inflammation and restenosis after percutaneous coronary interventions. Eur Heart J 2004;25:1679–87. 15451145 10.1016/j.ehj.2004.06.011

[8] Yahagi K , Kolodgie FD, Otsuka F, Finn AV, Davis HR, Joner M, Virmani R. Pathophysiology of native coronary, vein graft, and in-stent atherosclerosis. Nat Rev Cardiol 2016;13:79–98. 26503410 10.1038/nrcardio.2015.164

[9] Li J-a , Chen L, Zhang X-q, Guan S-k. Enhancing biocompatibility and corrosion resistance of biodegradable Mg-Zn-Y-Nd alloy by preparing PDA/HA coating for potential application of cardiovascular biomaterials. Mater Sci Eng C Mater Biol Appl 2020;109:110607. 32228927 10.1016/j.msec.2019.110607

[10] Zhang Z-Q , Yang Y-X, Li J-A, Zeng R-C, Guan S-K. Advances in coatings on magnesium alloys for cardiovascular stents – a review. Bioact Mater 2021;6:4729–57. 34136723 10.1016/j.bioactmat.2021.04.044 PMC8166647

[11] Durisin M , Reifenrath J, Weber CM, Eifler R, Maier HJ, Lenarz T, Seitz JM. Biodegradable nasal stents (MgF2 -coated Mg-2 wt %Nd alloy) – a long-term in vivo study. J Biomed Mater Res B Appl Biomater 2017;105:350–65. 26511430 10.1002/jbm.b.33559

[12] Costantino MD , Schuster A, Helmholz H, Meyer-Rachner A, Willumeit-Romer R, Luthringer-Feyerabend BJC. Inflammatory response to magnesium-based biodegradable implant materials. Acta Biomater 2020;101:598–608. 31610341 10.1016/j.actbio.2019.10.014

[13] Zhang H , Xie L, Shen X, Shang T, Luo R, Li X, You T, Wang J, Huang N, Wang Y. Catechol/polyethyleneimine conversion coating with enhanced corrosion protection of magnesium alloys: potential applications for vascular implants. J Mater Chem B 2018;6:6936–49. 32254578 10.1039/c8tb01574k

[14] Hsiao ST , Spencer T, Boldock L, Prosseda SD, Xanthis I, Tovar-Lopez FJ, Van Beusekom HM, Khamis RY, Foin N, Bowden N, Hussain A, Rothman A, Ridger V, Halliday I, Perrault C, Gunn J, Evans PC. Endothelial repair in stented arteries is accelerated by inhibition of Rho-associated protein kinase. Cardiovasc Res 2016;112:689–701. 27671802 10.1093/cvr/cvw210 PMC5157135

[15] Zanconato F , Forcato M, Battilana G, Azzolin L, Quaranta E, Bodega B, Rosato A, Bicciato S, Cordenonsi M, Piccolo S. Genome-wide association between YAP/TAZ/TEAD and AP-1 at enhancers drives oncogenic growth. Nat Cell Biol 2015;17:1218–27. 26258633 10.1038/ncb3216 PMC6186417

[16] Panciera T , Azzolin L, Cordenonsi M, Piccolo S. Mechanobiology of YAP and TAZ in physiology and disease. Nat Rev Mol Cell Biol 2017;18:758–70. 28951564 10.1038/nrm.2017.87 PMC6192510

[17] Lv Y , Kim K, Sheng Y, Cho J, Qian Z, Zhao YY, Hu G, Pan D, Malik AB, Hu G. YAP controls endothelial activation and vascular inflammation through TRAF6. Circ Res 2018;123:43–56. 29794022 10.1161/CIRCRESAHA.118.313143 PMC6014930

[18] Zhou X , Li W, Wang S, Zhang P, Wang Q, Xiao J, Zhang C, Zheng X, Xu X, Xue S, Hui L, Ji H, Wei B, Wang H. YAP aggravates inflammatory bowel disease by regulating M1/M2 macrophage polarization and gut microbial homeostasis. Cell Rep 2019;27:1176–89.e5. 31018132 10.1016/j.celrep.2019.03.028

[19] Wang L , Luo JY, Li B, Tian XY, Chen LJ, Huang Y, Liu J, Deng D, Lau CW, Wan S, Ai D, Mak KK, Tong KK, Kwan KM, Wang N, Chiu JJ, Zhu Y, Huang Y. Integrin-YAP/TAZ-JNK cascade mediates atheroprotective effect of unidirectional shear flow. Nature 2016;540:579–82. 27926730 10.1038/nature20602

[20] Wan S , Fu X, Ji Y, Li M, Shi X, Wang Y. FAK- and YAP/TAZ dependent mechanotransduction pathways are required for enhanced immunomodulatory properties of adipose-derived mesenchymal stem cells induced by aligned fibrous scaffolds. Biomaterials 2018;171:107–17. 29684675 10.1016/j.biomaterials.2018.04.035

[21] Yu H , He J, Su G, Wang Y, Fang F, Yang W, Gu K, Fu N, Wang Y, Shen Y, Liu X. Fluid shear stress activates YAP to promote epithelial-mesenchymal transition in hepatocellular carcinoma. Mol Oncol 2021;15:3164–83. 34260811 10.1002/1878-0261.13061 PMC8564657

[22] Schmidt D , Wilson MD, Spyrou C, Brown GD, Hadfield J, Odom DT. ChIP-seq: using high-throughput sequencing to discover protein-DNA interactions. Methods 2009;48:240–8. 19275939 10.1016/j.ymeth.2009.03.001 PMC4052679

[23] Guo F-X , Wu Q, Li P, Zheng L, Ye S, Dai X-Y, Kang C-M, Lu J-B, Xu B-M, Xu Y-J, Xiao L, Lu Z-F, Bai H-L, Hu Y-W, Wang Q. The role of the LncRNA-FA2H-2-MLKL pathway in atherosclerosis by regulation of autophagy flux and inflammation through mTOR-dependent signaling. Cell Death Differ 2019;26:1670–87. 30683918 10.1038/s41418-018-0235-z PMC6748100

[24] Bailey TL. DREME: motif discovery in transcription factor ChIP-seq data. Bioinformatics 2011;27:1653–9. 21543442 10.1093/bioinformatics/btr261 PMC3106199

[25] Hou Z , Xiang M, Chen N, Cai X, Zhang B, Luo R, Yang L, Ma X, Zhou L, He F, Yu H, Wang Y. The biological responses and mechanisms of endothelial cells to magnesium alloy. Regen Biomater 2021;8:rbab017. 34211729 10.1093/rb/rbab017 PMC8240605

[26] Bowen PK , Drelich J, Goldman J. A new in vitro-in vivo correlation for bioabsorbable magnesium stents from mechanical behavior. Mater Sci Eng C Mater Biol Appl 2013;33:5064–70. 24094225 10.1016/j.msec.2013.08.042

[27] Chinetti-Gbaguidi G , Colin S, Staels B. Macrophage subsets in atherosclerosis. Nat Rev Cardiol 2015;12:10–7. 25367649 10.1038/nrcardio.2014.173

[28] Monin L , Gaffen SL. Interleukin 17 family cytokines: signaling mechanisms, biological activities, and therapeutic implications. Cold Spring Harb Perspect Biol 2018;10:a028522. 28620097 10.1101/cshperspect.a028522 PMC5732092

[29] Seddon B. Thymic IL-7 signaling goes beyond survival. Nat Immunol 2015;16:337–8. 25789675 10.1038/ni.3128

[30] Yu FX , Zhao B, Guan KL. Hippo pathway in organ size control, tissue homeostasis, and cancer. Cell 2015;163:811–28. 26544935 10.1016/j.cell.2015.10.044 PMC4638384

[31] Piccolo S , Dupont S, Cordenonsi M. The biology of YAP/TAZ: hippo signaling and beyond. Physiol Rev 2014;94:1287–312. 25287865 10.1152/physrev.00005.2014

[32] Zaccara S , Ries RJ, Jaffrey SR. Reading, writing and erasing mRNA methylation. Nat Rev Mol Cell Biol 2019;20:608–24. 31520073 10.1038/s41580-019-0168-5

[33] Choe J , Lin S, Zhang W, Liu Q, Wang L, Ramirez-Moya J, Du P, Kim W, Tang S, Sliz P, Santisteban P, George RE, Richards WG, Wong KK, Locker N, Slack FJ, Gregory RI. mRNA circularization by METTL3-eIF3h enhances translation and promotes oncogenesis. Nature 2018;561:556–60. 30232453 10.1038/s41586-018-0538-8 PMC6234840

[34] Bonaa KH , Mannsverk J, Wiseth R, Aaberge L, Myreng Y, Nygard O, Nilsen DW, Klow NE, Uchto M, Trovik T, Bendz B, Stavnes S, Bjornerheim R, Larsen AI, Slette M, Steigen T, Jakobsen OJ, Bleie O, Fossum E, Hanssen TA, Dahl-Eriksen O, Njolstad I, Rasmussen K, Wilsgaard T, Nordrehaug JE, Investigators N. Drug-eluting or bare-metal stents for coronary artery disease. N Engl J Med 2016;375:1242–52. 27572953 10.1056/NEJMoa1607991

[35] Indolfi C , De Rosa S, Colombo A. Bioresorbable vascular scaffolds – basic concepts and clinical outcome. Nat Rev Cardiol 2016;13:719–29. 27681575 10.1038/nrcardio.2016.151

[36] Witte F , Ulrich H, Rudert M, Willbold E. Biodegradable magnesium scaffolds: part 1: appropriate inflammatory response. J Biomed Mater Res A 2007;81:748–56. 17390368 10.1002/jbm.a.31170

[37] Zhang B , Yao R, Li L, Li M, Yang L, Liang Z, Yu H, Zhang H, Luo R, Wang Y. Bionic tea stain-like, all‐nanoparticle coating for biocompatible corrosion protection. Adv Mater Interfaces 2019;6:1900899.

[38] Mao L , Shen L, Chen J, Zhang X, Kwak M, Wu Y, Fan R, Zhang L, Pei J, Yuan G, Song C, Ge J, Ding W. A promising biodegradable magnesium alloy suitable for clinical vascular stent application. Sci Rep 2017;7:46343. 28397881 10.1038/srep46343 PMC5387745

[39] Murakami S , Shahbazian D, Surana R, Zhang W, Chen H, Graham GT, White SM, Weiner LM, Yi C. Yes-associated protein mediates immune reprogramming in pancreatic ductal adenocarcinoma. Oncogene 2017;36:1232–44. 27546622 10.1038/onc.2016.288 PMC5322249

[40] Sorrentino G , Ruggeri N, Specchia V, Cordenonsi M, Mano M, Dupont S, Manfrin A, Ingallina E, Sommaggio R, Piazza S, Rosato A, Piccolo S, Del Sal G. Metabolic control of YAP and TAZ by the mevalonate pathway. Nat Cell Biol 2014;16:357–66. 24658687 10.1038/ncb2936

[41] Zhou Z , Hu T, Xu Z, Lin Z, Zhang Z, Feng T, Zhu L, Rong Y, Shen H, Luk JM, Zhang X, Qin N. Targeting Hippo pathway by specific interruption of YAP-TEAD interaction using cyclic YAP-like peptides. FASEB J 2015;29:724–32. 25384421 10.1096/fj.14-262980

[42] Zhang Z , Lin Z, Zhou Z, Shen HC, Yan SF, Mayweg AV, Xu Z, Qin N, Wong JC, Zhang Z, Rong Y, Fry DC, Hu T. Structure-based design and synthesis of potent cyclic peptides inhibiting the YAP-TEAD protein–protein interaction. ACS Med Chem Lett 2014;5:993–8. 25221655 10.1021/ml500160m PMC4160762

