# Supplementary material for: Yes-associated protein contributes to magnesium alloy-derivedinflammation in endothelial cells
Source: Regen Biomater. 2022 Jan 20;9:rbac002. doi: 10.1093/rb/rbac002 (PMC9039498; doi:10.1093/rb/rbac002)
Supplement: rbac002_Supplementary_Data [file rbac002_supplementary_data.pdf]

Supplementary information

Yes-associated Protein Contributes to Magnesium Alloy-derived Inflammation in Endothelial Cells

Hongchi Yu[1,3], Zhe Hou[1], Nuoya Chen[1], Rifang Luo[1], Li Yang[1], Michael Miao[4], Xiaoyi Ma[2], Lifeng Zhou[2], Fugui He[2], Yang Shen[3], Xiaoheng Liu[3*], Yunbing Wang[1*]
*: Corresponding authors

The supplementary materials are listed as below:

Supplemental Figure 1. Mg alloy promotes inflammatory genes expression.

Supplemental Figure 2. AZ-31 induces inflammatory response in tissue and HCAECs.

Supplemental Figure 3. The mRNA expression of transcriptional factors and co-transcriptional factors in inflammatory HCAECs.

Supplemental Figure 4. YAP modulates mRNA expression of inflammatory genes by binding to different DNA regions.

Supplemental Table 1. The inflammatory genes in heatmap.

Supplemental Table 2. Antibody list.

Supplemental Table 3. Silencing YAP target sequence.

Supplemental Table 4. Primer list.

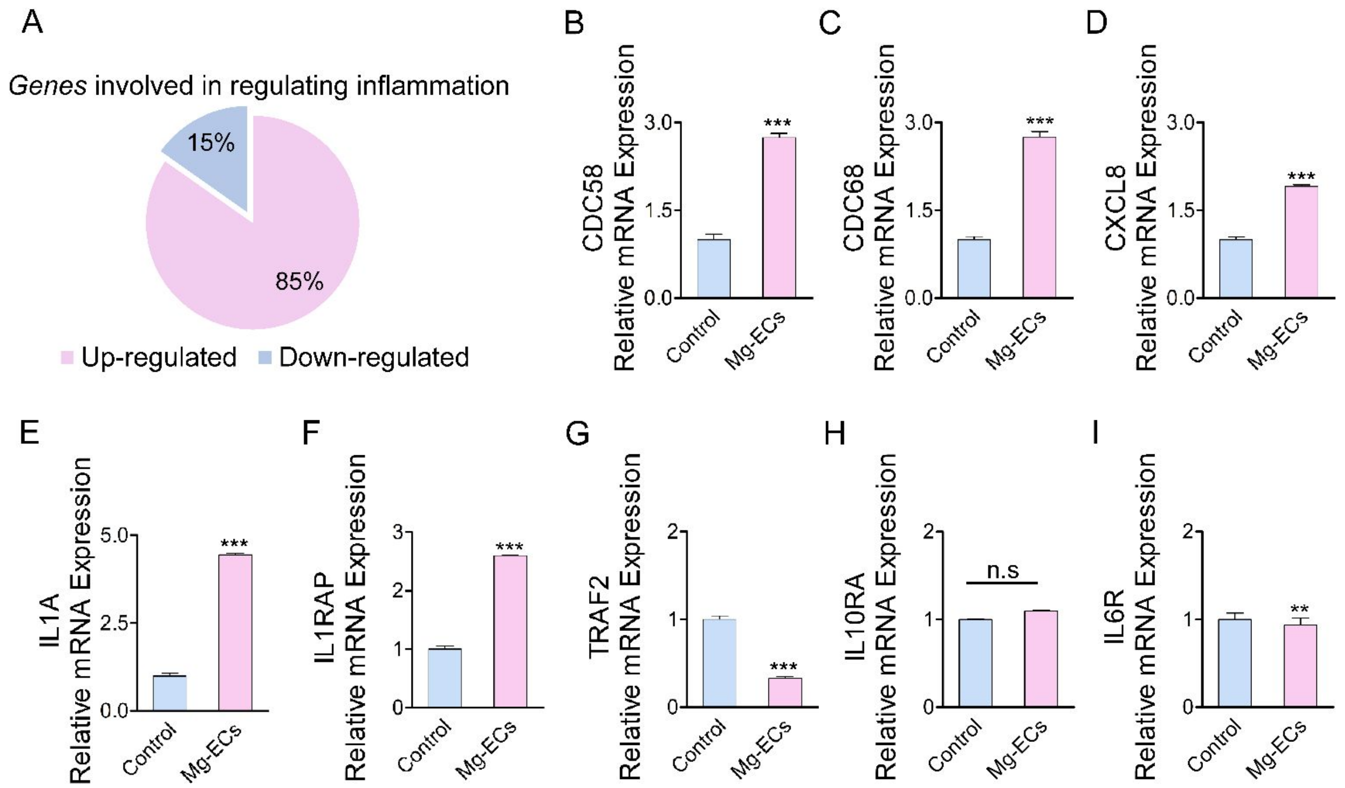

Supplemental Fig 1. Mg alloy promotes inflammatory genes expression. (**A**) The fraction of differently expressed genes associated with inflammation from mRNA-seq data. (**B-I**) The validation of genes related to inflammation from RNA-seq data was conducted by qRT-PCR analysis (n=3). Data are expressed as mean ± S.D, *P*-values were calculated according to two-tailed unpaired t-test, n.s means none significance, $^{**}P < 0.01$, $^{***}P < 0.001$.

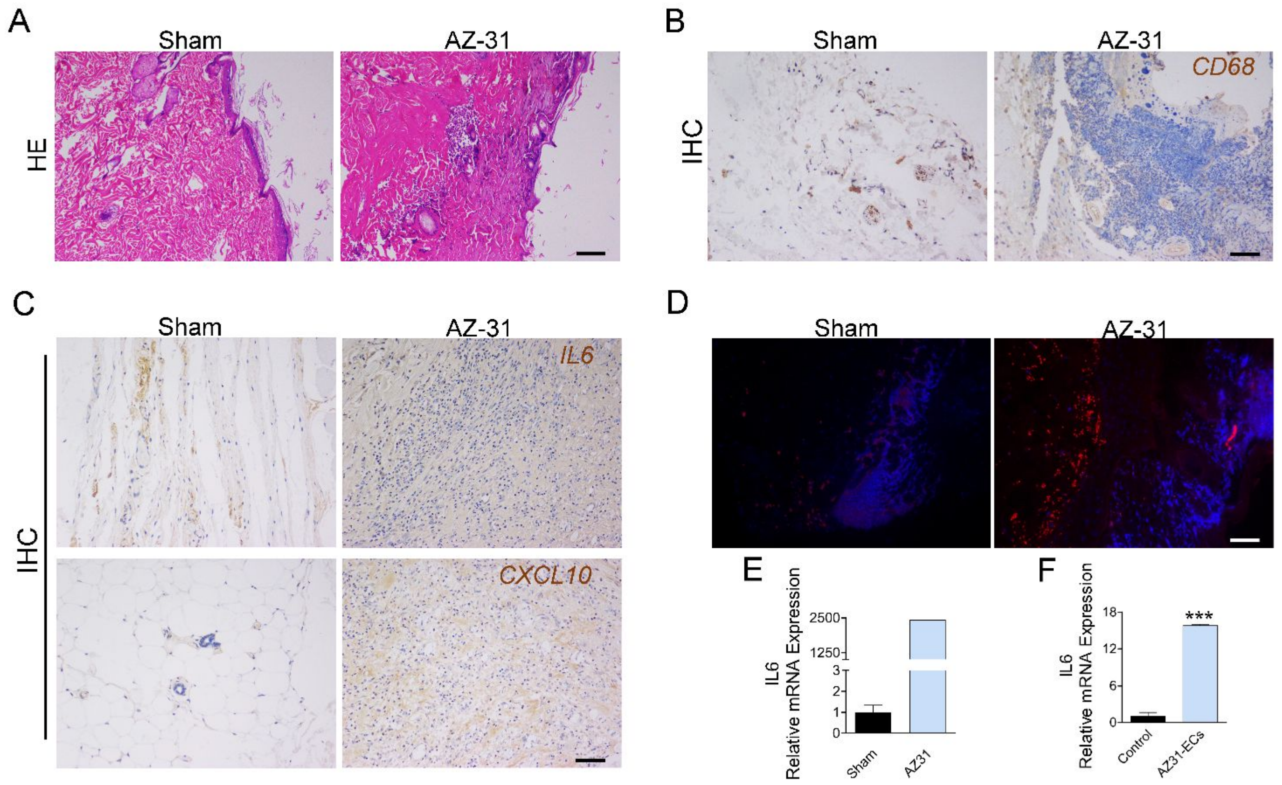

Supplemental Fig 2. AZ-31 induces an inflammatory response in tissue and HCAECs. (**A**) HE staining of subcutaneous tissue from rat with AZ-31 disc implantation, Scale bar, 50 µm. (**B**) Immunohistochemical staining of CD68 from subcutaneous tissue with AZ-31 disc implantation, Scale bar, 50 µm. (**C**) Immunohistochemical staining of IL6 and marker of Ⅰ-type macrophage CXCL10 from the tissue around AZ-31 disc at 2 weeks post-implantation, Scale bar, 50 µm. (**D**) Immunostaining showed higher expression of IL-6, Scale bar, 50 µm. (**E, F**) qRT-PCR analysis of *IL6* in subcutaneous tissue with AZ-31 disc implantation and AZ31-ECs (n=3). All the tissue and cell samples were obtained at 2 weeks after exposure to AZ-31. Data are shown as mean ± S.D, *P*-values were calculated according to a two-tailed unpaired t-test, $^{***}P < 0.001$.

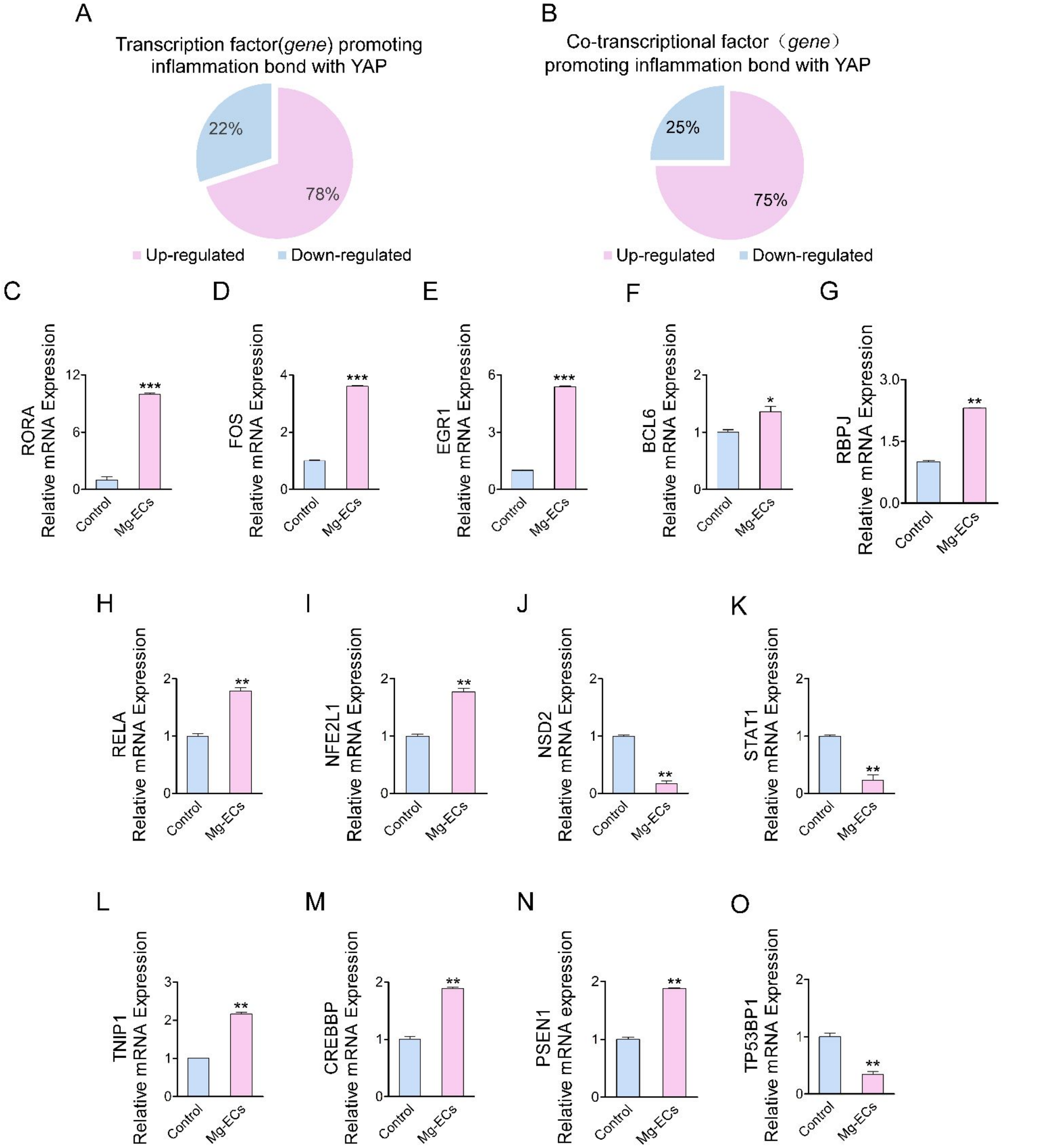

Supplemental Fig 3. The mRNA expression of YAP-targeted transcriptional factors and co-transcriptional factors in inflammatory HCAECs. (**A**) The fraction of YAP-targeted transcriptional factors related to inflammation after subjecting to Mg alloy is evaluated. (**B**) The fraction of YAP-targeted co-transcriptional factors related to inflammation after subjecting to Mg alloy is evaluated. (**C-K**) qRT-PCR analysis of YAP-targeted transcriptional factors related to inflammation. (**L-O**) qRT-PCR analysis of YAP-targeted co-transcriptional factors related to inflammation (n=3). Data are displayed as mean ± S.D, *P*-values were calculated according to a two-tailed unpaired t-test, $^{*}P < 0.05$, $^{**}P < 0.01$,$^{***}P < 0.001$.

A

Peak annotation	P-value	Regions classification
JAK1	0.000719	Exon
MIF	5.98E-05	Promoter
UCN	0.000974	Promoter
BMP2	0.000467	Enhancer
EDA2R	2.65E-05	Enhancer
CXCR4	0.000719	Enhancer
CCL20	7.49E-06	Enhancer
BMP6	0.000661	Enhancer

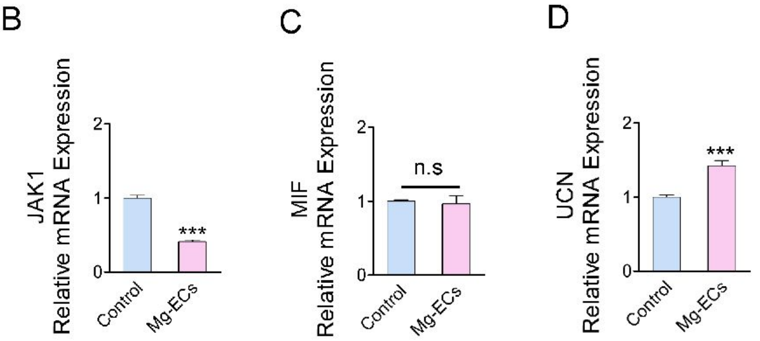

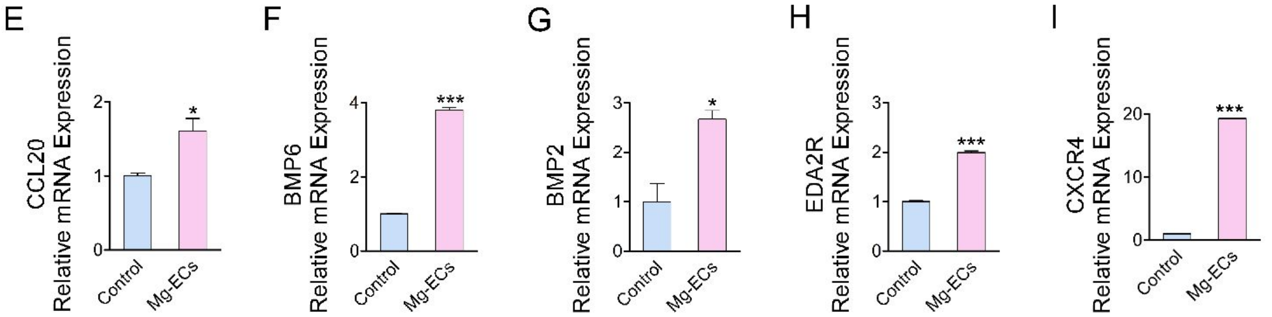

Supplemental Fig 4. YAP modulates mRNA expression of inflammatory genes by binding to different DNA regions. (**A**) The list of different DNA regions of several YAP-targeted inflammation genes. (**B-I**) qRT-PCR analysis of YAP-targeted inflammation genes. Data are presented as mean ± S.D, *P*-values were calculated according to a two-tailed unpaired t-test, $^{*}P < 0.05$, $^{**}P < 0.01$, $^{***}P < 0.001$.

Supplemental Tab 1. The inflammatory genes in heatmap

Gene Name	Fold Change (up-regulated)	Gene Name	Fold Change (up-regulated)
CRLF2	6.435038811	SOX9	2.456554422
APOL2	6.310943923	DSP	2.425041151
ALDOC	5.705210879	GBP2	2.419860553
SLC2A3	4.989788546	MUC3A	2.375606999
F3	4.646603618	CCL20	2.375038376
TNFRSF10D	3.979881311	PSAP	2.362875731
TNFAIP6	3.857849378	SFRP1	2.339136796
QSOX1	3.840587487	TNIP1	2.334500776
PLAUR	3.829120693	BMP2	2.333049062
BMP6	3.763419792	ACVR1	2.323221431
CXCR4	3.580352812	PTGES	2.316684381
EGR1	3.485375451	TNFRSF10B	2.288292913
MUC1	3.437202439	AMPD3	2.276531927
EEF1A1	3.367715404	ULBP2	2.261471512
LRP1	3.3153233	LGALS1	2.259963674
CD55	3.315237502	PKM	2.256902211
PRKCA	3.285778428	HDAC5	2.248254638
RBCK1	3.243351482	GRN	2.240760797
CDA	3.173551187	ATP6V0C	2.235992466
CD68	3.100880982	IL10RB	2.199822905
TCN1	3.055865313	AXL	2.19517765
C5AR1	3.0480488	IRAK2	2.168277403
UBC	3.032926946	SUSD4	2.161266695
IFI16	3.002469039	TGFB1	2.146994552
FOS	2.99895901	KDM6B	2.146551635
IRF7	2.937342758	SLAMF7	2.146033784
CTSD	2.909170734	ECM1	2.13289698
ALDOA	2.881677698	APP	2.120378437
NPC2	2.863207883	EDA2R	2.115442283
UBE2V1	2.820113892	BST2	2.099676688
GPI	2.769741768	PTGS2	2.095865476
NKX3-1	2.735755599	STBD1	2.087038784
CHST2	2.669641714	IFNE	2.07654283
CIB1	2.637667434	BCL10	2.072501718
HRH1	2.636921553	RARA	2.065234093
LGALS3	2.626668463	S100P	2.051367267
CDH17	2.555426533	KLF2	2.032606569
PRSS3	2.547352055	MUC20	2.025922468
ARHGEF2	2.5454548	TNIP2	2.024573814
CD58	2.531493324	BRI3	2.009689903
RELB	2.52797006	BTN3A2	2.007652257
TRPV1	2.499250749	IL1RAP	2.00274016
CXCL8	2.487224646	IST1	2.000452332
KRT16	2.465551571	ULBP1	1.992312946

NEU1	2.46045321	YPEL5	1.965163895
**Gene Name**	**Fold Change (up-regulated)**	**Gene Name**	**Fold Change (up-regulated)**
TRIM8	1.951042497	ANO6	1.653641977
DNASE1L1	1.942563365	ATP6V1D	1.651301219
F2RL1	1.923215927	GREM1	1.645635086
DOK3	1.920956894	DDRGK1	1.641239634
CLEC4E	1.909419174	ARL8A	1.638933714
IL1A	1.884755674	ITGAX	1.634521967
MANBA	1.867931287	ANPEP	1.629205454
RAB6A	1.867102109	C1RL	1.624629478
PLA2G4C	1.862752326	CTSA	1.623788094
CD63	1.859589381	IGBP1	1.603553448
ADORA2A	1.842497136	RBPJ	1.603013013
EEF2	1.83211164	TRIM52	1.595648684
CEBPG	1.829582079	KCMF1	1.586716242
CSTB	1.827360017	RHOG	1.585640034
CST3	1.825346978	TNFRSF10A	1.584607248
PNMA1	1.821343741	PDPK1	1.575350113
VAMP2	1.816926493	DNAJC3	1.563457994
IRAK1	1.810550539	TNFRSF11A	1.563332656
CCL5	1.803251536	DBNL	1.557454277
TIMP1	1.787132704	NFE2L1	1.55639106
ASAH1	1.781258702	TFPI	1.554310791
EXOSC6	1.78085968	CREBBP	1.545589218
TICAM1	1.780788099	MYD88	1.54214606
LAMTOR3	1.78074285	ARSA	1.539584372
PYGL	1.777414988	RELA	1.536147564
SPHK1	1.77264455	MILR1	1.534004181
PYGB	1.761451245	QPCT	1.532792084
MAPK14	1.755460825	AGER	1.53000457
S100A11	1.740039418	IKBKG	1.527893868
SDCBP	1.73833869	TSC1	1.526465065
GLIPR1	1.737518478	SPTAN1	1.52024451
MIF	1.734607864	HGSNAT	1.519670701
TMBIM1	1.730962681	RPS3	1.517730783
KMT2E	1.722250969	TRIM13	1.517624051
UCN	1.71310733	TERF2IP	1.516739086
SCN9A	1.712695257	RAP1B	1.515947636
FTH1	1.709199405	PSEN1	1.510164033
CSF2	1.708969	AOC3	1.509257745
TOM1	1.702341773	BCL6	1.506574449
MAP3K3	1.699488062	VAT1	1.503270119
CTSS	1.695364003	MAGT1	1.50264145
RAP2B	1.689029921		
LAMP2	1.676387148		
RORA	1.673659265		
TNFRSF10C	1.663937509		

PELI2	1.663490303		
**Gene Name**	**Fold Change (down-regulated)**	**Gene Name**	**Fold Change (down-regulated)**
EXO1	0.244680667	NECTIN2	0.529708285
STAT4	0.289057157	MLH1	0.538564813
RBP4	0.302323357	ADA	0.552659989
MSH2	0.318834913	IL6R	0.554371524
PRKDC	0.346939506	PTPN6	0.568468236
ATAD5	0.366322087	TRAF2	0.569655929
NSD2	0.372151638	NBN	0.569664921
UNG	0.406045097	PAXIP1	0.616380687
MSH6	0.409780137	MALT1	0.629773936
HMGB1	0.422201481	ZC3H12A	0.641091739
STAT1	0.451952901	HSPD1	0.643862194
DOCK10	0.463546909	HFE	0.645835646
GCNT3	0.475445944	TP53BP1	0.65941151
PRKCQ	0.510614209	RIF1	0.661083311
JAK1	0.514279154	EIF2AK4	0.664302909
NFKBID	0.516531299		

Supplemental Tab 2. Antibody list

Antibody information					Dilution/amount				
Antibody	Company	Catalog Number	Clone number	Description	Western Blot	ChIP-seq	ChIP-qPCR	Co-IP	IF
anti-β-actin （Human）	Cell Signaling Technology	#3700	8H10D10	Mouse monoclonal	1:1000				
anti-CDC42	Abcam	ab187643	EPR15620	Rabbit monoclonal	1:10000				1:200
anti-RhoA	Abcam	ab187027	EPR18134	Rabbit monoclonal	1:5000				1:150
anti-RAC1	Abcam	ab33186	23A8	Mouse monoclonal	1:1000				1:50
anti-Paxillin	Abcam	ab32084	Y113	Rabbit monoclonal	1:5000				1:200
anti-Vinculin	Abcam	ab129002	EPR8185	Rabbit monoclonal	1:10000				1:200
anti-YAP	Cell Signaling Technology	#14074	D8H1X	Rabbit monoclonal	1:1000				1:100
anti-YAP1	Abcam	ab52771	EP1674Y	Rabbit monoclonal		5ug/10^6 cells	5ug/10^6 cells		
anti-MST1	Cell Signaling Technology	#3682		Rabbit polyclonal	1:1000				
anti-LATS1	Cell Signaling Technology	#3477	C66B5	Rabbit monoclonal	1:1000				
anti-Ki-67	Cell Signaling Technology	#9449	8D5	Mouse monoclonal					1:800
anti-FAK	Abcam	ab40794	EP695Y	Rabbit monoclonal	1:1000				1:250
anti-p-FAK	Abcam	ab81298	EP2160Y	Rabbit monoclonal	1:1000				5μg/ml

Silencing YAP target sequence			
Clone Name	Location	Length	Target Sequence
shYAP21	857	21	GCTCAGCATCTTCGACAGTCT
shYAP22	920	21	GAGATGGCAAAGACATCTTCT
shYAP23	1051	21	GCAGAATATGATGAACTCGGC
shYAP24	1103	21	GCCATGACTCAGGATGGAGAA
TR001		19	GTTCGCGCCGTAGTCTTA

Supplemental Tab 3. Silencing YAP target sequence

Supplemental Tab 4. Primer list

List of primers used for RT-qPCR (RAT)			
Target transcript	Forward primer(5'-3')	Reverse primer(5'-3')	Product size (bp)
GAPDH(RAT)	GCTCTCTGCTCCTCCCTGTTCTA	TGGTAACCAGGCGTCCGATA	124
Lats1	TGATGCGGGTTGGATTAT	ATGCTCCTATTCCTAATGTCTT	144
Yap1	GACAACAACATGGCAGGACC	GGCTTGCTCCCATCCATC	133
Mst1	GACTTATCATCCCGAACAGA	CGACCTTGTTAATCCAGTCC	93
IL6	TCAGAGCAATACTGAAACCCTA	TCCTTAGCCACTCCTTCTGT	134
IL10	CTGCTATGTTGCCTGCTCT	ATGCTCCTTGATTTCTGGG	309

List of primers used for RT-qPCR (Human)			
Target transcript	Forward primer(5'-3')	Reverse primer(5'-3')	Product size (bp)
β-actin(Human)	GTGGCCGAGGACTTTGATTG	CCTGTAACAACGCATCTCATATT	73
GAPDH(Human)	GGGAAACTGTGGCGTGAT	GAGTGGGTGTCGCTGTTGA	299
CXCR4	CACGCCACCAACAGTCAGA	GACAATACCAGGCAGGATAAGG	225
CXCL8	CATACTCCAAACCTTTCCACC	ACTTCTCCACAACCCTCTGC	159
JAK1	CTGGGAAATCTGCTACAATGG	TGATGGCTCGGAAGAAAGG	176
IL6R	GCCAGTAGTGTCGGGAGCAA	CCATCCATGTTGTGAATGTCTTTG	226
TRAF2	GGGACCCTGAAAGAATACGAGA	CGCAGCCGTCACAAGTTAAG	232
CD58	ATACAGTGTACTCTTAGCAATCCAT	GTCACATTTCAGAATACCATTCATA	171
IL10RB	GGGTCGTGTGCTTGGAGG	CTGACATTTTCGGGAGGTGG	128
IL1RAP	AGAACAGAAGATGAAACAAGAACTC	TGCTTCACCTTGGCTGCT	131
CD68	CCTGCTTCTCTCATTCCCCTAT	GGACACATTGTACTCCACCGC	106
IL1A	TTCAAGGAGAGCATGGTGGT	TGAGGGCGTCATTCAGGAT	223
CCN2	CTTCGGTGGTACGGTGTA	GCTCTAATCATAGTTGGGTCT	279
AREG	CCCAAAACAAGACGGAAAGTG	ATGTTACTGCTTCCAGGTGCTC	177
CCL20	GCGCAAATCCAAAACAGAC	CCATTCCAGAAAAGCCACA	94
BMP2	CGTGCTTCTTAGACGGACTG	GCAGCAACGCTAGAAGACA	77
EDA2R	AGTTGTTCTCTCTGGACTCTGTTC	ATGCTCCAGATCAGTCCTTGT	154
BCL6	AGCCTGAGAACCTTGACC	CCCGTCATGGACCTGTTA	112
BMP6	GTCGTAATCGCTCTACCCAGTC	TGGTTGGTTGCATTCATGTG	223
MIF	GGCAAGCCCCCCCAGTAC	TGTCGTAATAGTTGATGTAGACCCTG	211
UCN	TCTGTCCATTGACCTCACCTTTC	CCATCACTTGCCCACCGA	121

MST1	CCCGTAGGGACAGGTTTCAC	CCGGAGCACTTGGAAGTCAT	162
LATS1	AGCACCTACACACCCTTCTTG	GTGGCATCACAGTCACATTTG	98
YAP1	GCCAGCAGGTTGGGAGAT	TGTGATTTAAGAAGTATCTCTGACC	59
CDC42	GATTACGACCGCTGAGTT	GAGTTATCTCAGGCACCC	111
RhoA	AAGATGGCATAACCTGTCTCA	TGGGAGGGAACCTGGATA	90
RAC1	GGAGACGGAGCTGTAGGT	AGTAGGGATATATTCTCCAGGA	78
PXN	GAGAAGCCTAAGCGGAATGG	AGATGCGTGTCTGCTGTTGG	175
VCL	CAGACCTGCTCCTTACCTTCG	AACTCTTTCACGGTGTTCATCG	244
TLN1	AAGGAGTATGTGAAGCAGAAGGGA	CAGCGTTTGATGTTGGTGAGGT	275
IL6	TGAGGAGACTTGCCTGGTGAA	GGTTGGGTCAGGGGTGGTTA	190
IL10	GCCCCTTGAGAAACCTTATTGT	GGCTTCTTTCTAAATCGTTCACAG	115
Mettl3	ACAGAGTGTCGGAGGTGATT	TGTAGTACGGGTATGTTGAGC	201
Mettl14	CCCATGTACTTACAAGCCGATAT	CCCCATTTTCGTAAACACACTC	257
Wtap	CCTCTTCCCAAGAAGGTTCGAT	GTTCCTTGGTTGCTAGTCGC	238
Fto	AATAGCCGCTGCTTGTGAG	CCACTTCATCTTGTCCGTTG	182
EGR1	CGAGCAGCCCTACGAGCAC	GGGAAAAGCGGCCAGTATAG	137
FOS	TTTGCCTAACCGCCACGAT3	CTGCGGGTGAGTGGTAGTAAGAG	120
RORA	TTCTTTCCCTACTGTTCGTTCA	CTCAAGTATTGGCAGGTTTCC	119
RBPJ	ATTCACAGTCCGAGATGGCT	GCGGTCTGCTTATCAACTTTC	114
NFE2L1	TTCCAGCATAACAGAAGCAGTG	CTGTAGTTGGTGCTCAGTGGGT	222
RELA	TGCCGAGTGAACCGAAAC	TCCCGTGAAATACACCTCAA	102
NSD2	CACGAAGCAGGAAGGGAGAT	GGCAAACTTGGTGTTGTAGCG	176
STAT1	TTTGCTGTATGCCATCCTCG	CACTGAGACATCCTGCCACCT	157
TNIP1	TGGAGGAGACCGACAAGGAG	CAAATGCTGTTGGTGGAGATG	195
CREBBP	CAGAACCAATGGAAGTGGATGA	CGAAGGAGATGTTGACTGAGAG	107
PSEN1	TCCACTTCGTATGCTGGTTGA	CAGGTGTAGAGCGATGAGGC	269
TP53BP1	TGTATCACCACAAGCCTCAAC	TCTCCATCTTTCTTCCCATCA	156

List of primers used for ChIP-PCR (Human)

Target transcript	Forward primer	Reverse primer	Product size (bp)
ANKRD1	AAAAAGGGCAGTGATGTGGTG	GAAGAGGGAGGGGAGGACAA	171
AMOTL2	TGCCAGGAATGTGAGAGTTTC	AGGAGGGAGCGGGAGAAG	108
CTGF	CTTTGGAGAGTTTCAAGAGCC	TCTGTCCACTGACATACATCC	150
IL10	GTAGGGATAGGTAAGAGGAAAGTA	TCTCCAGCACATAGAATGAAAC3	187
IL6	CAAGCCTGGGATTATGAAGAAGG	AGCACTGGCAGCACAAGGCAAAC	312
METTL3	CGTATTTGAAGACCTCTCGGAT	CAACGGTGGTCCCCAATCT	128
METTL14	GGGAAGAAGCCAAAAAATGC	ACGCAGGTAACAGCCACAAC	102
WTAP	CAGAGATGTCAAGGAGGAACGA	TGAGAACTTAGAAACCGATGCC	88
FTO	CATATCCTTGTGAGGCTGTTACTC	CATCCCGCTTCCTTGTTGT	152
RORA	TTAGTTTCTCCTCAGCCCTCG	TGAGATGATTCCGTGCCTACTT	158
RBPJ	GTGTAGCGTGAGACTGGACTGC	CGCCCGAAACCTGTTGAAG	97
NFE2L1	CACCTTCCTATTCCCTGACCT	GCCCTAGTGTCTGGCATTCT	152
RELA	GCCACTGGAGAAACTGAAATGA	CAGGCTCTGAAGAAACAGGAAG	78
BCL6	AGCAGTGGTAAAGTCCGAAGC	GCAATAATCACCTGGTGTCCG3	124
NSD2	GCCACAACTCAACCCATACAG3	CCACAGTTCACCTCCTCTTCC	88

STAT1	GTCTGGACCCTGACAACCTTA	CCTCTGGCATTCTTTCTACATT	75
TNIP1	TGACCTGGACCTGTTTATTTC	CTGAGTAGACCAAGGGATTTG	78
CREBBP	CAGGTAAAGACCGTGGGTAGAG	CTAAGCCAGAAACCCGTGAGT	79
PSEN1	CTCCCACAACTTATCTTTCATTC	CCTTCACATTCCTATCGCATC	161
TP53BP1	TCATTGGCTGCTGCCTGTC	CCCGCTCAAGTCTAAGTGGAA	168
EGR1	ACAGGTGGCAGAGGCAAGG	GCTGGGAAATTGAGGATAGGAA	85
FOS	AGACAGGAACTGCGAAATGCTC	GTGTAAACGTCACGGGCTCAA	157
